# Microarray-based gene set analysis: a comparison of current methods

**DOI:** 10.1186/1471-2105-9-502

**Published:** 2008-11-27

**Authors:** Sarah Song, Michael A Black

**Affiliations:** 1 Department of Statistics, University of Auckland, Auckland, New Zealand; 2 Department of Biochemistry, University of Otago, Dunedin, New Zealand

## Abstract

**Background:**

The analysis of gene sets has become a popular topic in recent times, with researchers attempting to improve the interpretability and reproducibility of their microarray analyses through the inclusion of supplementary biological information. While a number of options for gene set analysis exist, no consensus has yet been reached regarding which methodology performs best, and under what conditions. The goal of this work was to examine the performance characteristics of a collection of existing gene set analysis methods, on both simulated and real microarray data sets. Of particular interest was the potential utility gained through the incorporation of inter-gene correlation into the analysis process.

**Results:**

Each of six gene set analysis methods was applied to both simulated and publicly available microarray data sets. Overall, the various methodologies were all found to be better at detecting gene sets that moved from non-active (i.e., genes not expressed) to active states (or vice versa), rather than those that simply changed their level of activity. Methods which incorporate correlation structures were found to provide increased ability to detect altered gene sets in some settings.

**Conclusion:**

Based on the results obtained through the analysis of simulated data, it is clear that the performance of gene set analysis methods is strongly influenced by the features of the data set in question, and that methods which incorporate correlation structures into the analysis process tend to achieve better performance, relative to methods which rely on univariate test statistics.

## Background

Gene expression microarrays provide a snapshot of gene transcript abundance on a genomic scale, and are a popular tool for detecting differences in gene activity across biological samples. While many currently used approaches for analyzing microarray data focus on detach-detecting changes in activity on a per-gene basis [1,2], biological processes are generally the result of interactions between multiple genes (i.e., a gene pathway or a network), and are thus not easily interrogated via single gene methods. To facilitate such analyses, statistical methods have been developed which focus on detecting changes in groups of functionally related genes, thus allowing additional biological information to be incorporated into the analysis process [3-9]. Despite commonality of purpose, these methods take quite different approaches to achieving their goal, and can thus produce differing results when applied to the same data set. Recently Goeman [10] discussed the assumptions underlying these differing methodologies. Here we attempt to examine the performance differences between a number of methods through the analysis of real and simulated microarray data sets. In order to allow meaningful comparisons to be made, methods were selected for which software packages were available through the Bioconductor project [11], so that the ways by which gene sets were defined, and multiple comparisons corrections were performed could be strictly controlled, so as to ensure consistency across methods.

The basic goal of each of the methods investigated here is the same, to detect statistically significant changes in the expression activity of groups of genes. A necessary ingredient in the analysis process, therefore, is a collection of gene sets. These can be obtained in a number of ways. The first of these involves the use of publicly available annotation information such as that provided by the Kyoto Encyclopedia of Genes and Genomes (KEGG) [12], Gene Ontology [13], GenMAPP [14] and ResNet [15] databases. Taking this approach organizes genes into (possibly overlapping) groups which share common functional annotation, that is, sets of genes that are considered to be involved in the same underlying biological process. Although the assignment of genes to functional categories in this way is somewhat contentious (many factors influence the roles genes play in biological processes, so gene function is very much context-specific), it is currently (and will likely continue to be) a popular method for investigating the regulation of specific biological functions. While other methods for defining gene sets certainly exist (e.g., selecting highly correlated clusters of genes, independent of functional annotation), here we have chosen to employ a single, well-used approach, and focus on exploring the performance characteristics associated with each of the following analytic methods.

### Gene Set Enrichment Analysis (GSEA)

Currently the most well-known and widely used approach to gene set analysis, the GSEA method was introduced by Mootha *et al*. [3], and was used to identify pre-defined gene sets which exhibited significant differences in expression between samples from normal and diabetic patients. The methodology was subsequently refined by Subramanian *et al*. [4]. In this approach, genes are ranked by their signal-to-noise ratio (SNR) (the difference in means of the two class divided by the sum of the standard deviations of the two classes), and a "running sum" statistic is calculated for each gene set, based on the ranks of the members of the set, relative to those of the non-members. The maximum of this running sum across all genes is defined to be the "enrichment score" (ES). A high ES is achieved when a gene set contains a large number of highly ranked genes. A permutation-based *p*-value is then calculated for each gene set which is used to identify significant alterations in expression across experimental conditions. Bioconductor [11] includes two packages which implement the GSEA methodology, Category (implemented as specified in [16] with a modification to calculate the absolute value of per-gene test statistics) and limma [17], although there are some differences between the two, particularly in the way in which permutation-based *p*-values are calculated. In the Category package *p*-values are determined based on summing the *t*-statistics for the members of each gene set, while the limma package uses the mean of the *t*-statistics (i.e., essentially identical statistics, except for a scaling factor based on gene set size). In addition, the Category package computes *p*-values based on permutation of the sample labels, whereas the limma package uses permutation of gene labels. The relationship between these differing approaches to permutation and the underlying hypotheses being investigated are discussed in detail by Tian *et al*. [6], and by Goeman and Buhlmann [10], the latter of whom refer to tests as being either "self-contained" (samples permuted) or "competitive" (genes permuted).

### Significance Analysis of Function and Expression (SAFE)

The GSEA method was generalized and extended via the SAFE procedure [5] by taking a two-stage permutation-based approach to assessing significant changes in gene expression across experimental conditions. To accomplish this, the SAFE methodology utilizes the concept of *local *and *global *statistics. The local statistic measures the association between gene expression profiles and clinical outcomes, on a per-gene basis, generally using *t*-like statistics, or similar (essentially an alternative to the SNR employed by the original GSEA methodology [3]). The global statistic is then used to assess how the distribution of local statistics within a gene set differs to those outside the set. The Wilcoxon rank sum and Kolmogorov-Smirnov statistics are examples of possible global statistics, with the Wilcoxon rank sum statistic providing a similar setup to the original GSEA implementation [3]. Permutation of samples is then used to generate a *p*-value for the global statistic for each gene set. This methodology is implemented in the safe package, available through Bioconductor [11].

### Globaltest

The Globaltest methodology was introduced by Goeman *et al*. [7], and was designed to determine whether the common expression pattern of genes within a pre-defined set is significantly related to clinical outcome. Unlike the GSEA and SAFE procedures, no univariate per-gene statistics are utilized, instead a generalized linear model is used to estimate a "*Q*-statistic" for each gene set, which describes the correlation between gene expression profiles, *X*, and clinical outcomes, *Y*. The *Q*-statistic for a gene set is the average of the *Q*-statistics for each gene in the set. An inter-sample covariance matrix is used for calculation of the *Q*-statistic, which provides increased computational efficiency when testing gene sets that contain large numbers of genes.

### Principal Coordinates and Hotelling's T^2 ^(PCOT2)

This approach combines dimension reduction via Principal Coordinates [18] with Hotelling's multivariate extension of the *t*-test [19]. Versions of this approach were independently developed by Kong *et al*. [8] and Song *et al*. [20], with the pcot2 package [20] providing this functionality within Bioconductor. For each gene set, principal coordinates (PCO) is used to perform dimension reduction, allowing the possibly high-dimensional gene set data to be represented in low-dimensional space (e.g., two or three dimensions). This effectively creates a small number of meta-genes (linear combinations of the members of the gene set) which are assessed for changes in expression level across treatment conditions. The advantage of using PCO is that the inter-gene correlation structure is automatically incorporated into the process of dimension reduction, so this information is not lost. The use of Hotelling's T^2 ^procedure then allows this correlation (which is now represented as correlations between the lower dimensional meta-genes) to be included in the test statistic for each gene set. Like the Globaltest approach, the PCOT2 methodology generates a statistic for each gene set without the use of per-gene summaries.

### sigPathway

This methodology was developed by Tian *et al*. [6], and is used to test whether a group of genes is coordinately associated with a clinical outcome. Two possible null hypotheses to be tested are described in the paper: the first tests whether members of a gene set show the same pattern of association with a phenotype as non-members, while the second tests whether the expression patterns exhibited by members of a gene set are associated with the phenotype. As the second form of the null hypothesis reflects the default test used by all other gene set analysis methods investigated in this work (except for GSEA-limma), this was selected for use here. For each gene set a test statistic based on the average of the *t *statistics for each member gene is calculated. A permutation-based method is then used to generate the appropriate null distribution (genes for the first version of the null hypothesis, samples for the second version) for the test statistics, allowing *p*-values to be calculated. By default, the sigPathway approach uses a *q*-value approach to control the false discovery rate, however here the Benjamini and Yekutieli [21] correction was applied to the raw *p*-values produced by the calculate.NEk function so as to maintain consistency with the other methods used in this work. This methodology is implemented in the sigPathway package from Bioconductor [11].

### Gene set test statistics

Table 1 provides a summary of the default statistical methodology used to describe the changes observed in each gene set by these analysis methods, with *t *statistics providing the default per-gene summary for all methods employing univariate statistics. With the exception of the SAFE (which uses ranks of *t *statistics), all methods utilizing *t *statistics are essentially producing the same summary for each gene set, and thus any differences in performance must reflect differences in the approach for determining statistical significance of altered gene sets. The performance of each of these gene set analysis methods was assessed through the use of simulated data sets, the results of which are presented in the next section. In addition, two publicly available microarray data sets were analyzed using each of the methods.

**Table 1:** Summary of measurements used for each gene set method.

Method	Measurement of gene set	Permutation
GSEA-Category	The **sum **of the **T-statistics**	Sample
GSEA-limma	The **mean **of the **T-statistics**	Gene
SAFE	The **sum **of the **ranks **of the **T-statistics**	Sample
Globaltest	The **mean **of the **Q-statistics**	Sample
PCOT2	Hotelling's **T^2 ^**(multivariate T-statistic)	Sample
sigPathway	The **mean **of the **T-statistics**	Sample

## Results

### Performance on simple simulated data

The simulated data sets contained 40 microarrays, each consisting of 20 control and 20 treated samples. Four different types of gene set activity were simulated independently: (1) Off-Off (ND): Gene sets were inactive (i.e., genes within the group were not expressed) in both classes (control and treatment) with no difference (ND) in gene expression occurring between classes; (2)On-Off (D): Gene sets were active (i.e., genes co-expressed) in one class but not in the other, thus generating a difference (D) between the means of the genes in the set, across the two classes; (3) On-On (ND): Gene sets were active in both classes but with no difference (ND) in expression activity; (4) On-On (D): Gene sets were active in both classes, with a difference (D) between the means of the member genes, across the two classes. In this simulation a total of 400 genes were used, spread across 20 non-overlapping gene sets, with 20 genes per gene set.

Gene set activity was simulated in the following proportions for each data set: 0.4 Off-Off (ND), 0.2 On-Off (D), 0.2 On-On (ND) and 0.2 On-On (D), with the multivariate normal distribution used to generate data from active (ON) gene sets (to allow co-expression, and hence correlation), and a standard normal distribution (mean 0, standard deviation 1) used to generate data from inactive (OFF) gene sets (no expression, so no correlation possible).

The results of applying each of the six gene set analysis methods to a collection of 100 of these small simulated microarray data sets are presented in Table 2, where the values in each cell relate to the proportion of gene sets exhibiting of each type of expression activity [On-Off (D), On-On (D), Off-Off (ND), On-On (ND)] that were correctly identified by each of the methods. For the simulation an inter-mean difference of 0.5 was used in conjunction with a pairwise correlation between genes of 0.1. For each method 10,000 permutations were used to generate *p*-values for each gene set. The results show that the GSEA-Category, Globaltest and PCOT2 methods were able to detect more of the gene sets exhibiting altered expression [On-Off (D) and On-On (D)] than the SAFE, GSEA-limma and sigPathway methods were, at a cost of reporting slightly more false positives, corresponding to lower proportions of correctly identified Off-Off (ND) and On-On (ND) gene sets.

**Table 2:** Detection rates for the six gene set analysis methods on simple simulated data.

	PCOT2	SAFE	GSEA-Category	GSEA-limma	Globaltest	sigPathway
On-Off (D)	0.902	0.236	0.912	0.24	0.93	0.002
	(0.013)	(0.021)	(0.012)	(0.019)	(0.011)	(0.002)
On-On (D)	0.858	0.22	0.85	0.268	0.886	0
	(0.016)	(0.019)	(0.014)	(0.019)	(0.014)	(0)
Off-Off (ND)	0.994	1	0.996	1	0.992	0.998
	(0.003)	(0)	(0.003)	(0)	(0.005)	(0.002)
On-On (ND)	0.99	1	0.994	1	0.992	1
	(0.004)	(0)	(0.003)	(0)	(0.004)	(0)

100 data sets (each containing 20 genes in 20 sets) were analyzed by each method, with 10,000 permutations used to generate *p*-values to which FDR controlling adjustments [21] were made. An adjusted *p*-value of 0.05 was required for significance. The value in each cell relates to the proportion of each type of gene set activity correctly identified by each method. Standard errors are shown in parentheses.

**Figure 1:**
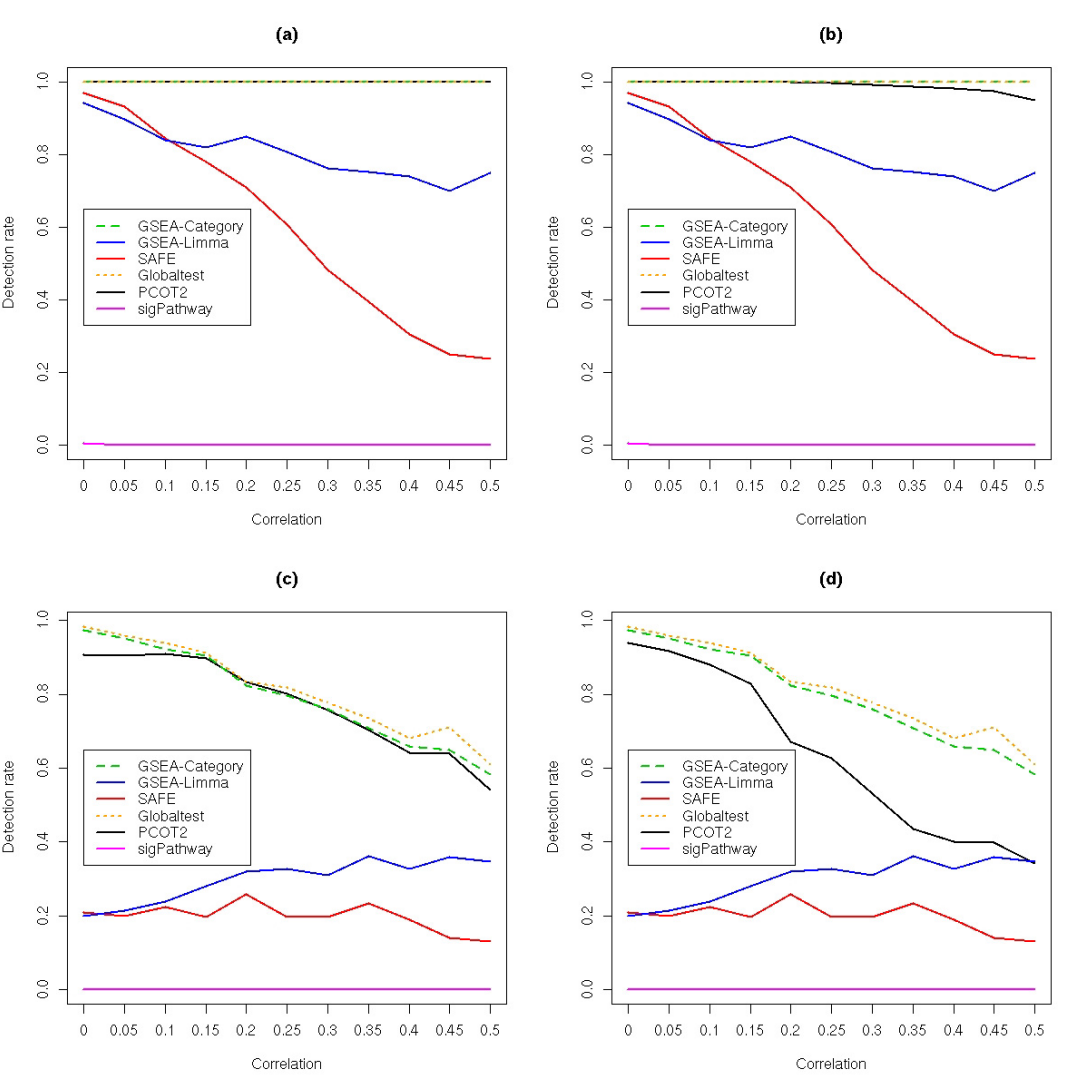
**Detection rates in simulated data sets as a function of increasing correlation**. For each gene set analysis method, the data were permuted 2,000 times to generate *p*-values for each gene set, within each simulation. FDR-adjusted *p*-values of less than 0.05 were used to indicate significance. (a) Gene sets active in one class and inactive in the other, On-Off (D), with a difference between the means of 1. (b) Gene sets active in both classes, On-On (D), with a difference between the means of 1. (c) Gene sets active in one class and inactive in the other, On-Off (D), with a difference between the means of 0.5. (d) Gene sets active in both classes, On-On (D), with a difference between the means of 0.5.

Figure 1 presents the detection rate for active gene sets as a function of increasing inter-gene correlations, for two different inter-class separations (inter-mean differences of 1 or 0.5), with 2,000 permutation used to generate *p*-values for each gene set. For well-separated classes (panels (a) and (b), difference of 1), the GSEA-Category, Globaltest and PCOT2 methods all performed consistently well across the range of correlations, while the GSEA-limma and SAFE methods exhibited a drop in performance with increasing inter-gene correlation. For a smaller separation between the classes (panels (c) and (d), difference of 0.5), the performance of GSEA-Category, Globaltest and PCOT2 decreased with increasing correlation, but the performance of the GSEA-limma and PCOT2 methods actually appeared to increase, although the detection ability of these methods was still much lower than that of the top three. The performance of the sigPathway method was extremely poor on the simulated data, with this approach unable to detect any altered pathways at these levels of separation between the classes. Further investigation of this revealed that the directionality of the changes observed within the gene sets was a major factor in this poor performance. When the simulation was repeated using gene sets where all altered genes had the same direction of change (e.g.., all up-regulated), the performance of the sigPathway method improved markedly. This suggests that the test statistic generated for each gene set requires consistent changes in expression by member genes, otherwise the opposing changes tend to cancel each other out. These results are presented in Additional file 1.

**Figure 2:**
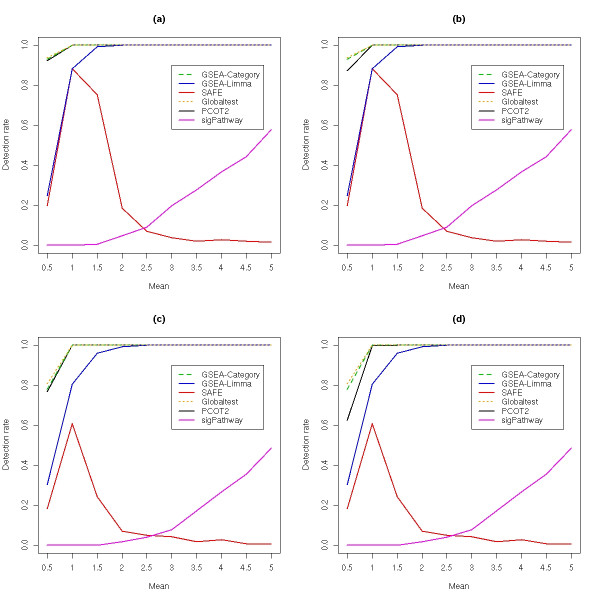
**Detection rates in simulated data sets as a function of increasing inter-class separation**. For each gene set analysis method, the data were permuted 2,000 times to generate *p*-values for each gene set, within each simulation. FDR-adjusted *p*-values of less than 0.05 were used to indicate significance. (a) Gene sets active in one class and inactive in the other, On-Off (D), with pairwise correlations of 0.1. (b) Gene sets were active in both classes, On-On (D), with pairwise correlations of 0.1. (c) Gene sets active in one class and inactive in the other, On-Off (D), with pairwise correlations of 0.25. (d) Gene sets were active in both classes, On-On (D), with pairwise correlations of 0.25.

An interesting relationship amongst the six methods is observed in Figure 2, which shows the effect of increasing the amount of separation between the classes for fixed levels of correlation (pairwise correlations of either 0.1 or 0.25), with 2,000 permutations again used to generate gene set *p*-values. While both GSEA methods, along with Globaltest, PCOT2 and sigPathway showed improved performance with increasing inter-class separation, the SAFE procedure actually exhibited worse performance. While seemingly strange, it must be remembered that SAFE operates on the ranks of genes, which do not take the magnitude of the inter-class differences into account. The sigPathway method can be seen to perform relatively poorly at even moderate to high degrees of inter-class separation, however, this again appears to be the result of genes exhibiting non-consistent changes (in terms of direction) within the gene sets.

### Simulations based on microarray data

Each of the six gene set analysis methods were next used to analyze a more complex collection of simulated data sets, the structure of which (in terms of inter-class separation, and inter-gene correlation) was based on that of two publicly available microarray data sets, one relating to diabetes [3], and the other to leukemia [22]. These data sets were used to provide realistic values for the parameters *p*, *μ *and Σ in (1). Distributions of the average *μ *and Σ (in terms of pairwise correlations) values for each gene set are shown in Additional files 2 and 3, for the two data sets utilized.

The first data set was the diabetes data published by Mootha *et al*. [3] with the introduction of the GSEA methodology. These data contain expression information on 22,283 genes in skeletal muscle biopsy samples from 43 age-matched males: 17 with normal glucose tolerance (NGT), 9 with impaired glucose tolerance (IGT) and 17 with type 2 diabetes mellitus (DM2). In the analysis presented here, only the 34 samples relating to the NGT and DM2 classes were used. In addition to the microarray data, Mootha *et al*. [3] also provide the 150 gene sets used in the initial GSEA analysis (available on-line at ). These lists were used to define the gene sets used in the analyses presented here, so that all six methods produced results based on exactly the same collection of gene sets.

The second microarray data set used was the leukemia data originally published by Golub *et al*. [22]. This data consists of 34 samples: 20 from patients with acute lymphoblastic leukemia (ALL) and 14 from patients with acute myeloid leukemia (AML). These data are publicly available as the golubEsets Bioconductor package [11]. For the leukemia data, the Bioconductor annotation package hu6800 was used to define 134 gene sets relating to KEGG pathways  used in the analysis. As for the diabetes data, the same collection of gene sets was used by each of the six analysis methods. Variance stabilization [23] was applied to the data using the vsn Bioconductor package so as to maintain consistency with a previous gene set analysis of these data [24].

As both data sets utilized the Affymetrix Genechip technology, a number of genes were represented by multiple probe sets, potentially giving these genes more weight in the analysis. To avoid this problem, the probe set median was calculated for any gene represented by more than one probe set. To restrict attention to gene sets of a reasonable size, gene sets with less than 10 members were excluded from the analysis. This resulted in the diabetes data set retaining 3728 genes in 87 gene sets, and the leukemia data retaining 2383 genes in 127 gene sets.

For each data set, values were obtained (*p*) or estimated (*μ*, Σ) for each gene set, and 100 data sets were simulated using (1), with each then analyzed using the six gene set analysis methods.

Table 3 contains the results of applying each of the gene set analysis methods to these simulated data sets. This approach to simulation resulted in a major difference in the degree of inter-class separation across the two data sets, with the simulated gene sets based on the diabetes data exhibiting far smaller changes in expression than those based on the leukemia data (reflecting the different structure of these data sets). As a result there was a substantial drop in the ability of all methods to detect changes in gene sets that were active in both classes in the diabetes data, as compared to the leukemia data. In both cases, however, all methods were more successful at detecting changes relating to gene sets switching from being inactive to active between classes, than detecting expression alterations in gene sets that were active in both classes. Across both simulations, the two methods that incorporate correlation information (Globaltest and PCOT2) correctly identified the most differentially expressed gene sets, which suggests that accounting for correlation is an important issue in gene set analyses.

**Table 3:** Detection rates for the six gene set analysis methods on realistic simulated data sets.

*Simulations based on diabetes data *[3]						
	PCOT2	SAFE	GSEA-Category	GSEA-limma	Globaltest	sigPathway

On-Off [D]	0.997	0.637	1	0.587	1	0.773
	(0.003)	(0.031)	(0)	(0.019)	(0)	(0.024)
On-On [D]	0.07	0.007	0.063	0	0.087	0.047
	(0.013)	(0.005)	(0.015)	(0)	(0.017)	(0.012)
Off-Off [ND]	0.993	1	0.997	1	0.997	0.997
	(0.003)	(0)	(0.002)	(0)	(0.002)	(0.002)
On-On [ND]	0.997	1	1	1	0.997	1
	(0.003)	(0)	(0)	(0)	(0.003)	(0)

*Simulations based on leukemia data *[22]						

	PCOT2	SAFE	GSEA-Category	GSEA-limma	Globaltest	sigPathway

On-Off [D]	1	0.384	0.998	0.552	1	0.966
	(0)	(0.031)	(0.002)	(0.021)	(0)	(0.007)
On-On [D]	0.5	0	0.444	0	0.584	0.18
	(0.023)	(0)	(0.022)	(0)	(0.023)	(0.017)
Off-Off [ND]	0.997	1	0.998	1	0.99	0.995
	(0.001)	(0)	(0.001)	(0)	(0.003)	(0.002)
On-On [ND]	0.994	1	0.99	1	0.99	0.996
	(0.003)	(0)	(0.004)	(0)	(0.004)	(0.003)

100 data sets were simulated, with gene numbers and gene set membership determined by data-specific values derived from the diabetes [3] and leukemia [22] data sets. The simulated data sets were analyzed by each gene set analysis method, with 2,000 permutations used to generate *p*-values to which FDR controlling adjustments were made. An adjusted *p*-value of 0.05 was required for significance. The value in each cell relates to the proportion of each type of gene set activity correctly identified by each method. Standard errors are shown in parentheses.

### Analysis of microarray data

**Figure 3:**
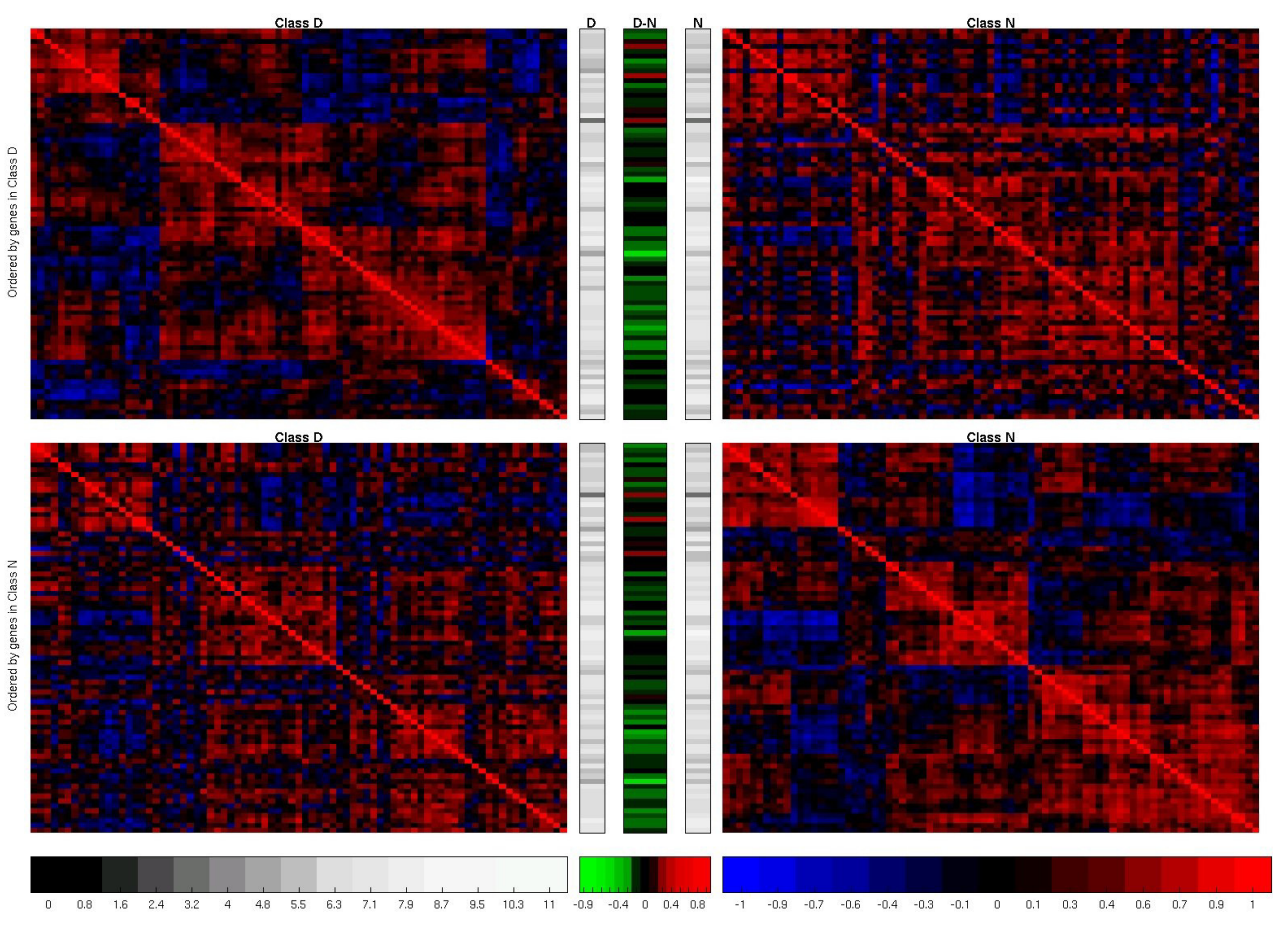
**Visualization of expression and correlation in the OXPHOS HG-U133A probes pathway using the pcot2 package**. The four red-blue plots represent pairwise correlations between genes in the pathway, with positively correlated genes clustered together. The top left plot relates to the inter-gene correlations observed within the DM2 samples, while the top right plot contains the inter-gene correlation information for the NGT samples, *with genes in the same order as the top left plot *(i.e., the gene order is the same in both plots on the top row). The same approach is taken in the bottom two plots, with the bottom right plot representing inter-gene correlation within the NGT samples, with genes again grouped by correlation. The gene order in the bottom left plot (DM2 samples) is then the same as that in the bottom right. The gray-scale plots in the center of the figure indicate gene expression intensity, while the red-green plots show the change in expression level (red indicates up-regulation in DM2 relative to NGT).

In order to investigate the similarity (in terms of gene sets detected as having significantly altered expression) across approaches, the six gene set analysis methods were applied to each of the two publicly available microarray data sets described above, the diabetes data set analyzed by Mootha *et al*. [3] and the leukemia data set of Golub *et al*. [22]. The results for the diabetes data are presented in Table 4 and Additional file 4. As in the original analysis of this data, the Oxidative Phosphorylation gene set (OXPHOS_HG-U133A_probes) was identified by both implementations of the GSEA procedure as being the gene set most likely to have undergone a change in expression activity. Interestingly, however, GSEA-limma was the only approach that reported this change to be significant after correction for multiple testing. The most likely explanation for this is that the GSEA-limma methodology defaults to permuting genes in the calculation of *p*-values, and is essentially testing a different null hypothesis (as described by Tian et al. [6]). The significance noted here, therefore, can be interpreted to mean that the genes contains in the OXPHOS_HG-U133A_probes gene set are significantly more different across classes than genes that are not members of that gene set, but that the degree of separation between the classes could still have been observed by chance (based on the non-significance of the GSEA-Category *p*-value). Figure 3 contains a graphical summary of the gene activity relating to the Oxidative Phosphorylation gene set, and was produced using the functionality of the pcot2 Bioconductor package [20]. Information on intra-class correlation, absolute expression, and differential expression is presented. Here it can be observed that a subset of genes within the OXPHOS_HG-U133A_probes gene set exhibit tightly correlated expression patterns within the NGT class, and that most of these probes are down-regulated in the DM2 class (with a subsequent decrease in correlation).

**Table 4:** Application of gene set analysis methods to diabetes data [3].

Top Pathways	NP	AP
*GSEA-Category*		
OXPHOS_HG-U133A_probes	0.071	1
MAP00480_Glutathione_metabolism	0.091	1
MAP00500_Starch_and_sucrose_metabolism	0.19	1
MAP00252_Alanine_and_aspartate_metabolism	0.199	1
GLUCO_HG-U133A_probes	0.202	1
......	...	...

*GSEA-limma*		
OXPHOS_HG-U133A_probes	<0.001	<0.001
c18_U133_probes	0.004	0.88
Human_mitoDB_6_2002_HG-U133A_probes	0.006	0.88
Mitochondr_HG-U133A_probes	0.01	1
MAP00190_Oxidative_phosphorylation	0.018	1
......	...	...

*SAFE*		
MAP00561_Glycerolipid_metabolism	0.011	1
OXPHOS_HG-U133A_probes	0.021	1
MAP00500_Starch_and_sucrose_metabolism	0.035	1
MAP00240_Pyrimidine_metabolism	0.046	1
GLUCO_HG-U133A_probes	0.049	1
......	...	...

*GlobalTest*		
MAP00252_Alanine_and_aspartate_meta...	0.106	1
OXPHOS_HG-U133A_probes	0.123	1
c23_U133_probes	0.14	1
c25_U133_probes	0.144	1
GLUCO_HG-U133A_probes	0.144	1
......	...	...

*PCOT2*		
c20_U133_probes	0.032	1
OXPHOS_HG-U133A_probes	0.045	1
MAP00190_Oxidative_phosphorylation	0.06	1
FA_HG-U133A_probes	0.086	1
c28_U133_probes	0.093	1
......	...	...

*sigPathway*		
c22_U133_probes	0.012	1
c29_U133_probes	0.014	1
OXPHOS_HG-U133A_probes	0.014	1
c25_U133_probes	0.032	1
MAP00252_Alanine_and_aspartate_metabolism	0.088	1
......	...	...

Top five altered gene sets determined by each of the six analysis methods. NP indicates the nominal *p*-values and AP indicates the FDR adjusted *p*-values.

**Figure 4:**
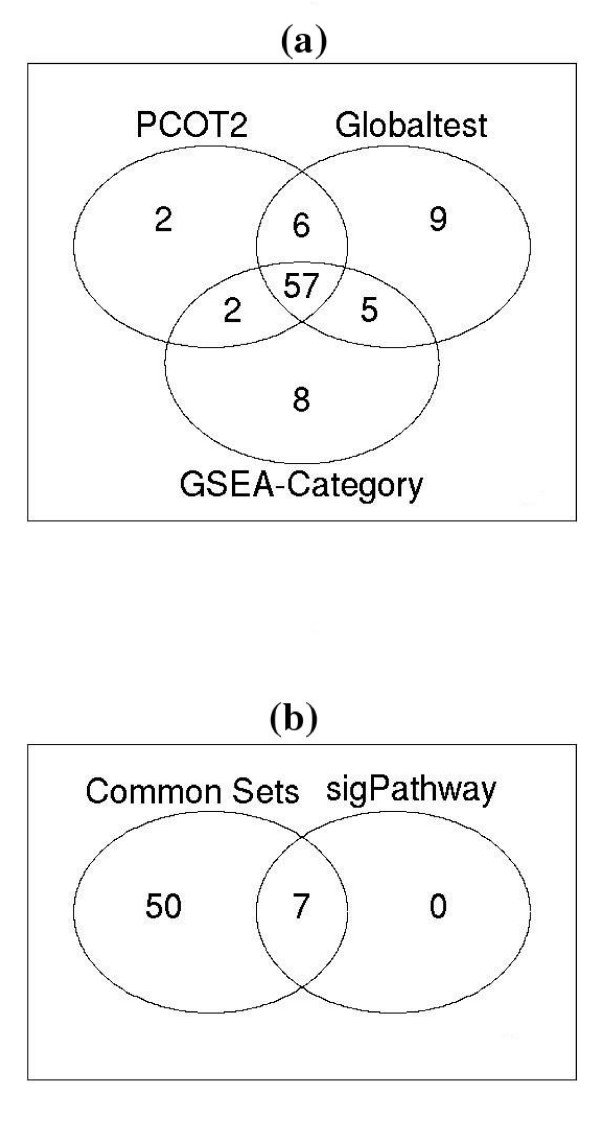
**Significant gene sets detected in the leukemia data set (GSEA-Category, Globaltest, PCOT2, sigPathway)**. (a) The GSEA-Category, Globaltest and PCOT2 approaches detected 72 (GSEA-Category), 77 (Globaltest) and 67 (PCOT2) gene sets as undergoing significant changes in expression activity, after correction for multiple testing, with 57 gene sets detected as significantly altered by all three approaches. The two methods which incorporate correlation structure into their assessment procedure (Globaltest and PCOT2) exhibited strong agreement in the gene sets they found to be altered (63 significant gene sets in common). (b) Of the 57 changed gene sets identified in common by GSEA-Category, Globaltest and PCOT2, 7 were also found by sigPathway.

The results for the leukemia data are presented in Table 5 and Additional file 5. Based on the adjusted *p*-values, all methods except SAFE were able to identify gene sets with significantly altered expression activity. The remaining approaches reported 2 (GSEA-limma), 72 (GSEA-Category), 67 (PCOT2), 77 (Globaltest) and 7 (sigPathway) significantly altered gene sets. The overlap between the latter four of these methods is presented in Figure 4, with 57 gene sets detected in common by three approaches (GSEA-Category, PCOT2 and Globaltest). Of these 57, 7 were also detected by sigPathway, mirroring the results of the simulation analysis in terms of the similarity of performance between these approaches. There is also a high level of agreement between the Globaltest and PCOT2 methods, with 63 gene sets detected in common, which most likely reflects the similarity of these approaches (in terms of the incorporation of correlation information), and again reinforces the close agreement observed in the simulated data sets. In order to further explore the lower detection rate of the sigPathway approach, the expression patterns of the genes within the 57 common gene sets were examined. Within the 7 gene sets detected by sigPathway, on average, 71% of genes exhibited altered expression in the same direction, whereas in the remaining 50 gene sets the average was only 58%, further suggesting that the Bioconductor implementation of sigPathway requires members of altered gene sets to exhibit changes in a consistent direction.

**Table 5:** Application of gene set analysis methods to leukemia data [22].

Top Pathways	NP	AP
*GSEA-Category*		
Glycolysis/Gluconeogenesis	<5e-05	<5e-05
Focal adhesion	<5e-05	<5e-05
Tight junction	<5e-05	<5e-05
Leukocyte transendothelial migration	<5e-05	<5e-05
Regulation of actin cytoskeleton	<5e-05	<5e-05
......	...	...

*GSEA-limma*		
Hematopoietic cell lineage	<5e-05	0.034
B cell receptor signaling pathway	<5e-05	0.034
Glutathione metabolism	0.017	1
Glycolysis/Gluconeogenesis	0.025	1
Natural killer cell mediated cytotoxicity	0.028	1
......	...	...

*SAFE*		
Natural killer cell mediated cytotoxicity	0.0052	1
Glycolysis/Gluconeogenesis	0.00835	1
Galactose metabolism	0.0128	1
Pyrimidine metabolism	0.0333	1
Cell cycle	0.0353	1
......	...	...

*GlobalTest*		
Toll-like receptor signaling pathway	<5e-05	<5e-05
Jak-STAT signaling pathway	<5e-05	<5e-05
Focal adhesion	<5e-05	<5e-05
Tight junction	<5e-05	<5e-05
Leukocyte transendothelial migration	<5e-05	<5e-05
......	...	...

*PCOT2*		
Jak-STAT signaling pathway	<5e-05	0.001
Glycolysis Gluconeogenesis	<5e-05	0.001
Focal adhesion	<5e-05	0.001
Tight junction	<5e-05	0.001
Hematopoietic cell lineage	<5e-05	0.001
......	...	...

*sigPathway*		
Arachidonic acid metabolism	<5e-05	0.001
Metabolism of xenobiotics by cytochrome P450	<5e-05	0.004
Glutathione metabolism	<5e-05	0.009
Cell cycle	0.0001	0.016
Starch and sucrose metabolism	0.0002	0.023
......	...	...

Top five altered gene sets determined by each of the six analysis methods. NP indicates the nominal *p*-values and AP indicates the FDR adjusted *p*-values.

## Discussion

Gene set analysis has become an important aspect of the analysis and interpretation of gene expression microarray data. Despite the availability of a number of different gene set analysis methodologies, there is currently very little information on their relative merits. Here we have investigated the performance characteristics of a subset of commonly used approaches through the analysis of both simulated and real microarray data. A recent paper by Liu et al. [9] used a similar approach to investigate the performance of three different gene set analysis methods, one of which was the version of Globaltest implemented here. The simulated data generated in that publication were also based on a multivariate normal distribution, although parameters were specified by the authors (as in the first simulation presented here), rather than being based on estimates derived from real microarray data sets (as in the second simulation in this work). The main finding of the work by Liu *et al*. [9] was that the results achieved by the three approaches investigated were broadly similar, particularly when standardization methods were applied to the data. In contrast, for the methodologies examined here, we found substantial differences in performance between approaches in both the simulation studies that were performed. The most likely reason for these differences relates to the gene set analysis methods that were investigated. The study by Liu *et al*. [9] compared the performance of two methods based on the Globaltest methodology (Global Test, and ANCOVA Global Test [25]), and one (SAM-GS [26]) that was very similar (in that it utilized a t-like statistic followed by sample permutation) to the implementation of GSEA in the category package that was used here. Given the high concordance between Globaltest and GSEA-Category that was observed in this study, it is reassuring that the three methods presented by Liu *et al*. [9] gave good agreement with one another. In addition, the supplementary data provided in that paper included performance data for a competitive (i.e., permutation of genes) version of GSEA that exhibited lower power than Globaltest. The same relationship was observed here when comparing Globaltest to the GSEA-limma methods, which also involved gene permutation.

Based on our results, we feel that of the six approaches examined, the GSEA (as implemented in the Category package [16]), Globaltest and PCOT2 methodologies all exhibited similar performance in the situations explored here, with the correlation-based methods (Globaltest and PCOT2) offering slightly better performance in some settings. The fact that the GSEA-Category approach was able to perform well in the presence of inter-gene correlation (despite it not explicity using this information), most likely reflects the relatively low within-class correlation levels that were observed. That is, the Globaltest and PCOT2 approaches did not seem to gain a major advantage through their use of correlation structures in the settings explored here.

The SAFE methodology, the limma implementation of GSEA, and the sigPathway method, emerged as performing relatively poorly in this analysis. For the SAFE methodology, this is due to the rank-based system employed by default in the safe package, which fails to take the magnitude of the separation between classes into account. For gene sets containing a collection of genes for which a large degree of separation is present between classes, permutation will still tend to result in these genes having highly ranked t-statistics, as many permutations will still produce a substantial degree of interclass separation, due to the large difference between the class means. Because of this, gene sets exhibiting large differences across classes can still produce large test statistics under permutation when a rank-based method is used to summarize gene set activity, whereas a statistic that does not use ranks (e.g., taking the mean of the per-gene t-statistics for a gene set) will be much less likely to produce a permutation-based test statistic of the same magnitude as that generated from the original data.

For the GSEA-limma approach, the choice to permute genes rather than samples in the calculation of *p*-values for each gene set appears to be the dominant difference between this method and that of the GSEA-Category implementation, and is therefore highly likely to explain the difference in performance. The decision on whether to permute rows or columns of the data matrix obviously reflects a difference in the null hypothesis being investigated, and must therefore be considered by individual researchers. For the sigPathway method, our additional investigations have revealed that consistency in the direction of expression changes is required for the detection of significantly altered gene sets, and this can largely explain the lower detection rate exhibited by this approach.

The incorporation of correlation into the analytic process by the Globaltest and PCOT2 approaches reflects a step forward in gene set analysis methodologies. Correlation has recently "re-emerged" as an important measure of inter-gene relatedness, and forms the basis for the very active field of regulatory network construction [27-29]. The ability to incorporate information about likely co-regulation (rather than simple co-expression) into the analysis process serves as a useful surrogate for functional relatedness, and thus adds to the biological interpretability of the results. It also raises the possibility of defining gene sets by observed (within-class) correlation structure, rather than by functional relationship (as defined by public databases). Such an approach allows the data to drive the grouping of genes (as in the early days of microarray data analysis, where hierarchical clustering often used to assign genes to groups exhibiting similar expression patterns), which can then be tested for changes in expression across classes, before being mapped back to functional classes, thus avoiding a bias in gene set construction induced by the database information (which is often skewed towards commonly investigated genes, tissues, organisms and diseases). With the presentation of these results, we hope that researchers will now have an understanding of the relative performance of some of the commonly used gene set analysis methods. To further this understanding, as additional procedures are developed, we encourage other authors to include comparative simulation data of the type provided here to contrast the performance of newly proposed methodologies with existing gene set analysis techniques.

## Methods

### Data sets

In order to introduce an inter-gene correlation structure into the simulated data, the multivariate normal distribution with mean *μ *and variance-covariance matrix Σ was used to generate the data for each gene set, based on the following density function:

(1) $$f(x)=\frac{1}{{\left(2\pi \right)}^{p/2}|\Sigma {|}^{1/2}}e^{-(x-\mu {)}^{\prime }\Sigma^{-1}(x-\mu )/2}$$

where X = [*x*_1_, *x*_2_, ⋯, *x*_*p*_] is a *p *× 1 vector, and each element of X is a random variable with the multivariate normal distribution with dimensionality *p*. The correlation matrix *ρ *can be obtained from the variance-covariance matrix Σ by *ρ *= (*V*^1/2^)^-1^Σ(*V*^1/2^)^-1 ^where *V *is the *p *× *p *variance matrix of variable *X*. In the simulated data, the variance-covariance matrix was equal to the correlation matrix, as the diagonal entries in the variance-covariance matrix were all equal to 1.

### P-value calculation and multiple comparisons procedures

Each of the gene set analysis methods are able to produce *p*-values which are uncorrected for multiplicity (i.e., nominal or unadjusted *p*-values), based on the use of permutation of either samples or genes. Here the default permutation approach employed by each method was used, except in the case of sigPathway (which returns results for both sample and gene permutation) where the outputs relating to sample permutation were utilized. In order to remove any variability introduced through the use of different multiple comparisons corrections, the False Discovery Rate (FDR) controlling method of Benjamini and Yekutieli [21] was used to provide control of Type I errors when testing for changes in multiple gene sets. This method of *p*-value adjustment was applied to the nominal *p*-values produced by all of the gene set analysis methods. For the small simulated data sets, 10,000 permutations were used to generate *p*-values, while for both the second (more complex) simulated data sets and Mootha's microarray data set, 2000 permutations were used. For Golub's data, 20,000 permutations were applied to produce more finely grained *p*-values so as to better differentiate top-ranked significantly changed pathways.

A FDR controlling approach was favored over more traditional methods of family-wise error rate control, as FDR-based methodology is currently the most commonly used approach for microarray analyses. The choice of the Benjamini-Yekutieli FDR controlling method [21] reflects the fact that correlation may exist between gene sets, and thus test statistics, either because two gene sets are involved in the same biological process, or because individual genes appear in multiple gene sets. For all hypothesis tests, an adjusted *p*-value of less than 0.05 was considered to indicate a statistically significant change in gene set expression.

### Implementation

All gene set analysis methods used in this paper are available as software packages through the Bioconductor project .

## Authors' contributions

All data analysis was performed by SS. The manuscript was prepared by SS and MAB. Both authors have read and approved the final version of the manuscript.

## Supplementary Material

Additional file 1 **Detection rates for the six gene set analysis methods on simple simulated data**. In contrast to Table 2, all altered gene sets were simulated so as to exhibit changes in the same direction. This resulted in a major performance improvement for the sigPathway approach. 100 data sets (each containing 20 genes in 20 sets) were analyzed by each method, with 10,000 permutations used to generate *p*-values to which FDR controlling adjustments [21] were made. An adjusted *p*-value of 0.05 was required for significance. The value in each cell relates to the proportion of each type of gene set activity correctly identified by each method. Standard errors are shown in parentheses. Click here for file

Additional file 2 **The distribution of difference and pairwise correlations in diabetes data**[**3**]. (a) difference for each gene, positive value indicates up-regulation in DM2 samples (b) average difference for each gene set (c) pairwise correlations for all gene pairs using DM2 samples (d) average pairwise correlations for each gene set using DM2 samples (e) pairwise correlations for all pairs of genes using NGT samples (f) average pairwise correlations for each gene set using NGT samples. Click here for file

Additional file 3 **The distribution of difference and pairwise correlations in the leukemia data**[**22**]. (a) difference for each gene, positive value indicates up-regulation in AML samples (b) average difference for each gene set (c) pairwise correlations for all gene pairs using AML samples (d) average pairwise correlations for each gene set using AML samples (e) pairwise correlations for all pairs of genes using ALL samples (f) average pairwise correlations for each gene set using ALL samples. Click here for file

Additional file 4 **Application of gene set analysis methods to diabetes data**[**3**]**(all gene sets)**. Ranked (by *p*-value) gene sets produced by each of the six analysis methods. NP indicates the nominal *p*-values and AP indicates the FDR adjusted *p*-values. Click here for file

Additional file 5 **Application of gene set analysis methods to leukemia data**[**22**]**(all gene sets)**. Ranked (by *p*-value) gene sets produced by each of the six analysis methods. NP indicates the nominal *p*-values and AP indicates the FDR adjusted *p*-values. Click here for file
