# Supplementary material for: Microarray-based gene set analysis: a comparison of current methods
Source: BMC Bioinformatics. 2008 Nov 27;9:502. doi: 10.1186/1471-2105-9-502 (PMC2607289; doi:10.1186/1471-2105-9-502)
Supplement: Additional file 1 — Detection rates for the six gene set analysis methods on simple simulated data. In contrast to Table 2, all altered gene sets were simulated so as to exhibit changes in the same direction. This resulted in a major performance improvement for the sigPathway approach. 100 data sets (each containing 20 genes in 20 sets) were analyzed by each method, with 10,000 permutations used to generate p-values to which FDR controlling adjustments [21] were made. An adjusted p-value of 0.05 was required for significance. The value in each cell relates to the proportion of each type of gene set activity correctly identified by each method. Standard errors are shown in parentheses. [file 1471-2105-9-502-S1.pdf]

Additional file 3 - Detection rates for the six gene set analysis methods on simple simulated data.

100 data sets (each containing 20 genes in 20 sets) were analyzed by each method, with 10,000 permutations used to generate p-values to which FDR controlling adjustments were made. An adjusted p-value of 0.05 was required for significance. The value in each cell relates to the proportion of each type of gene set activity correctly identified by each method. Standard errors are shown in parentheses.

	PCOT2	SAFE	GSEA-Category	GSEA-Limma	Globaltest	sigPathway
On-Off (D)	0.876	0.19	0.892	0.28	0.922	0.988
	(0.015)	(0.021)	(0.015)	(0.02)	(0.014)	(0.005)
On-On (D)	0.746	0.11	0.746	0.274	0.802	0.868
	(0.019)	(0.014)	(0.021)	(0.018)	(0.017)	(0.014)
Off-Off (ND)	0.992	1	0.994	1	0.994	0.992
	(0.004)	(0)	(0.003)	(0)	(0.003)	(0.005)
On-On (ND)	0.996	1	0.994	1	0.992	0.994
	(0.003)	(0)	(0.003)	(0)	(0.004)	(0.003)
