# Supplementary figures and images for: Microarray-based gene set analysis: a comparison of current methods
Source: BMC Bioinformatics. 2008 Nov 27;9:502. doi: 10.1186/1471-2105-9-502 (PMC2607289; doi:10.1186/1471-2105-9-502)

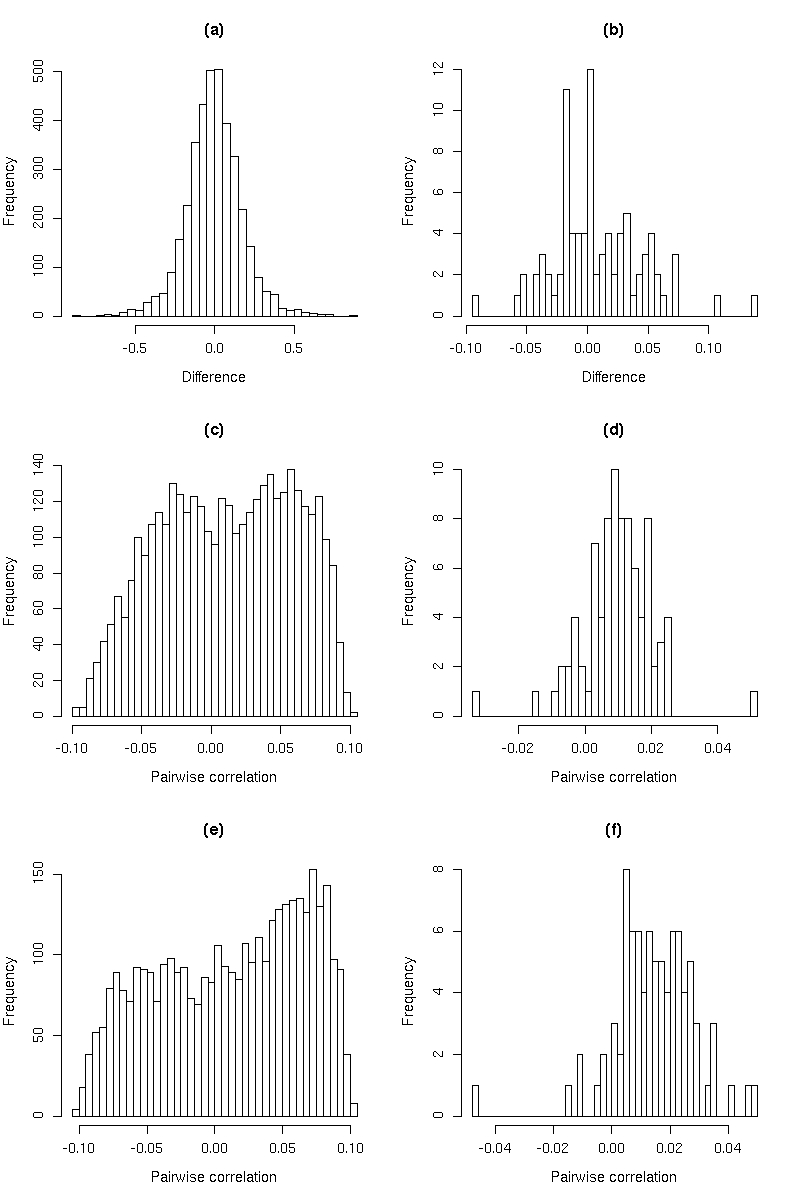

Supplement: Additional file 2 — The distribution of difference and pairwise correlations in diabetes data[3]. (a) difference for each gene, positive value indicates up-regulation in DM2 samples (b) average difference for each gene set (c) pairwise correlations for all gene pairs using DM2 samples (d) average pairwise correlations for each gene set using DM2 samples (e) pairwise correlations for all pairs of genes using NGT samples (f) average pairwise correlations for each gene set using NGT samples. [file 1471-2105-9-502-S2.jpeg]

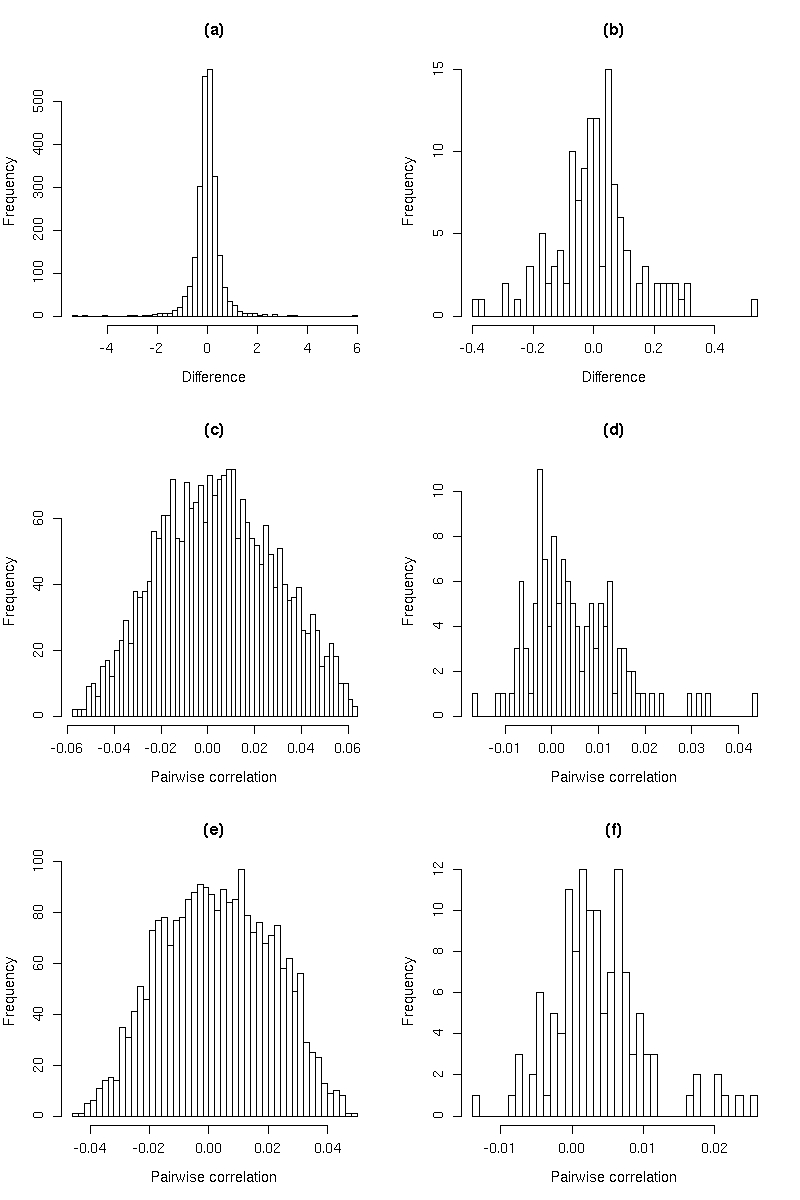

Supplement: Additional file 3 — The distribution of difference and pairwise correlations in the leukemia data[22]. (a) difference for each gene, positive value indicates up-regulation in AML samples (b) average difference for each gene set (c) pairwise correlations for all gene pairs using AML samples (d) average pairwise correlations for each gene set using AML samples (e) pairwise correlations for all pairs of genes using ALL samples (f) average pairwise correlations for each gene set using ALL samples. [file 1471-2105-9-502-S3.jpeg]
