# Supplementary material for: Microarray-based gene set analysis: a comparison of current methods
Source: BMC Bioinformatics. 2008 Nov 27;9:502. doi: 10.1186/1471-2105-9-502 (PMC2607289; doi:10.1186/1471-2105-9-502)
Supplement: Additional file 4 — Application of gene set analysis methods to diabetes data[3](all gene sets). Ranked (by p-value) gene sets produced by each of the six analysis methods. NP indicates the nominal p-values and AP indicates the FDR adjusted p-values. [file 1471-2105-9-502-S4.pdf]

Additional file 1 - The full table of results on Mootha's data set. Ranked (by p-value) gene sets produced by each of the five analysis methods. NP indicates the nominal p-values and AP indicates the FDR adjusted p-values.

Top Pathways	NP	AP
GSEA-Category		
OXPHOS_HG-U133A_probes	0.071	1
MAP00480_Glutathione_metabolism	0.091	1
MAP00500_Starch_and_sucrose_metabolism	0.19	1
MAP00252_Alanine_and_aspartate_metabolism	0.199	1
GLUCO_HG-U133A_probes	0.202	1
MAP00561_Glycerolipid_metabolism	0.237	1
MAP00190_Oxidative_phosphorylation	0.251	1
MAP00240_Pyrimidine_metabolism	0.298	1
c23_U133_probes	0.303	1
c18_U133_probes	0.341	1
MAP00710_Carbon_fixation	0.378	1
MAP00251_Glutamate_metabolism	0.392	1
MAP00860_Porphyrin_and_chlorophyll_metabolism	0.395	1
MAP00052_Galactose_metabolism	0.398	1
MAP00670_One_carbon_pool_by_folate	0.41	1
c20_U133_probes	0.418	1
MAP00220_Urea_cycle_and_metabolism_of_amino_groups	0.443	1
MAP00030_Pentose_phosphate_pathway	0.445	1
MAP00510_N_Glycans_biosynthesis	0.458	1
MAP00910_Nitrogen_metabolism	0.47	1
c31_U133_probes	0.473	1
c3_U133_probes	0.477	1
human_mitoDB_6_2002_HG-U133A_probes	0.486	1
MAP03020_RNA_polymerase	0.503	1
FA_HG-U133A_probes	0.504	1
mitochondr_HG-U133A_probes	0.514	1
c21_U133_probes	0.52	1
c14_U133_probes	0.528	1
c2_U133_probes	0.53	1
c8_U133_probes	0.534	1
MAP00590_Prostaglandin_and_leukotriene_metabolism	0.535	1
c25_U133_probes	0.543	1
MAP00230_Purine_metabolism	0.545	1
c30_U133_probes	0.554	1
c10_U133_probes	0.557	1
TCA_HG-U133A_probes	0.568	1
MAP00330_Arginine_and_proline_metabolism	0.578	1
c22_U133_probes	0.606	1
c16_U133_probes	0.608	1

Continue ... ...

Top Pathways	NP	AP
c1_U133_probes	0.608	1
c33_U133_probes	0.614	1
c24_U133_probes	0.619	1
INS_HG-U133A_probes	0.622	1
c26_U133_probes	0.625	1
GLYCOL_HG-U133A_probes	0.65	1
c27_U133_probes	0.666	1
MAP00120_Bile_acid_biosynthesis	0.679	1
MAP00310_Lysine_degradation	0.681	1
MAP00340_Histidine_metabolism	0.69	1
c11_U133_probes	0.727	1
GLYCOGEN_HG-133A_probes	0.742	1
MAP00193_ATP_synthesis	0.751	1
MAP00195_Photosynthesis	0.751	1
MAP03070_Type_III_secretion_system	0.751	1
c7_U133_probes	0.752	1
MAP00970_Aminoacyl_tRNA_biosynthesis	0.753	1
MAP00350_Tyrosine_metabolism	0.767	1
c4_U133_probes	0.789	1
c35_U133_probes	0.792	1
MAP00620_Pyruvate_metabolism	0.794	1
c34_U133_probes	0.803	1
c13_U133_probes	0.819	1
MAP00051_Fructose_and_mannose_metabolism	0.82	1
c29_U133_probes	0.828	1
MAP00020_Citrate_cycle_TCA_cycle	0.83	1
c15_U133_probes	0.836	1
c19_U133_probes	0.873	1
MAP00562_Inositol_phosphate_metabolism	0.881	1
MAP00071_Fatty_acid_metabolism	0.893	1
c9_U133_probes	0.895	1
MAP00640_Propanoate_metabolism	0.908	1
GO_0005739_HG-U133A_probes	0.91	1
c32_U133_probes	0.913	1
MAP00361_gamma_Hexachlorocyclohexane_degradation	0.921	1
c28_U133_probes	0.934	1
c0_U133_probes	0.938	1
c17_U133_probes	0.949	1
MAP00410_beta_Alanine_metabolism	0.95	1
MAP00010_Glycolysis_Gluconeogenesis	0.954	1
c6_U133_probes	0.96	1
MAP00280_Valine_leucine_and_isoleucine_degradation	0.962	1
c5_U133_probes	0.969	1
c12_U133_probes	0.985	1
	Continue	

Top Pathways	NP	AP
MAP00650_Butanoate_metabolism	0.987	1
MAP00260_Glycine_serine_and_threonine_metabolism	0.989	1
MAP00380_Tryptophan_metabolism	0.993	1
MAP00380_Tryptophan_metabolism	0.993	1
GSEA-limma		
OXPHOS_HG-U133A_probes	<0.001	<0.001
c18_U133_probes	0.004	0.879
human_mitoDB_6_2002_HG-U133A_probes	0.006	0.879
mitochondr_HG-U133A_probes	0.01	1
MAP00190_Oxidative_phosphorylation	0.018	1
c20_U133_probes	0.028	1
c23_U133_probes	0.042	1
MAP00561_Glycerolipid_metabolism	0.048	1
GLUCO_HG-U133A_probes	0.048	1
MAP00500_Starch_and_sucrose_metabolism	0.048	1
MAP00252_Alanine_and_aspartate_metabolism	0.055	1
MAP00240_Pyrimidine_metabolism	0.063	1
c3_U133_probes	0.067	1
c21_U133_probes	0.12	1
MAP00710_Carbon_fixation	0.128	1
c10_U133_probes	0.162	1
MAP00251_Glutamate_metabolism	0.17	1
MAP00860_Porphyrin_and_chlorophyll_metabolism	0.171	1
MAP00030_Pentose_phosphate_pathway	0.172	1
MAP00052_Galactose_metabolism	0.186	1
MAP00230_Purine_metabolism	0.192	1
MAP00910_Nitrogen_metabolism	0.206	1
FA_HG-U133A_probes	0.207	1
MAP03020_RNA_polymerase	0.21	1
MAP00220_Urea_cycle_and_metabolism_of_amino_groups	0.212	1
c26_U133_probes	0.216	1
c33_U133_probes	0.216	1
MAP00510_N_Glycans_biosynthesis	0.219	1
c1_U133_probes	0.243	1
c25_U133_probes	0.248	1
c22_U133_probes	0.252	1
MAP00330_Arginine_and_proline_metabolism	0.256	1
GLYCOL_HG-U133A_probes	0.276	1
MAP00310_Lysine_degradation	0.346	1
MAP00340_Histidine_metabolism	0.354	1
c27_U133_probes	0.358	1
MAP03070_Type_III_secretion_system	0.362	1
GLYCOGEN_HG-133A_probes	0.362	1

Continue ... ...

Top Pathways	NP	AP
MAP00193_ATP_synthesis	0.375	1
c7_U133_probes	0.378	1
MAP00120_Bile_acid_biosynthesis	0.379	1
MAP00195_Photosynthesis	0.39	1
MAP00620_Pyruvate_metabolism	0.406	1
MAP00970_Aminoacyl_tRNA_biosynthesis	0.416	1
MAP00051_Fructose_and_mannose_metabolism	0.424	1
MAP00020_Citrate_cycle_TCA_cycle	0.428	1
c13_U133_probes	0.491	1
c29_U133_probes	0.491	1
c34_U133_probes	0.494	1
c35_U133_probes	0.504	1
MAP00410_beta_Alanine_metabolism	0.521	1
MAP00071_Fatty_acid_metabolism	0.545	1
MAP00010_Glycolysis_Gluconeogenesis	0.546	1
c9_U133_probes	0.554	1
MAP00650_Butanoate_metabolism	0.556	1
c15_U133_probes	0.565	1
c19_U133_probes	0.575	1
c5_U133_probes	0.58	1
MAP00260_Glycine_serine_and_threonine_metabolism	0.586	1
GO_0005739_HG-U133A_probes	0.592	1
c32_U133_probes	0.595	1
MAP00380_Tryptophan_metabolism	0.626	1
MAP00380_Tryptophan_metabolism	0.628	1
MAP00280_Valine_leucine_and_isoleucine_degradation	0.63	1
MAP00562_Inositol_phosphate_metabolism	0.632	1
MAP00361_gamma_Hexachlorocyclohexane_degradation	0.64	1
MAP00640_Propanoate_metabolism	0.642	1
c0_U133_probes	0.642	1
c17_U133_probes	0.664	1
c12_U133_probes	0.664	1
c6_U133_probes	0.671	1
c28_U133_probes	0.68	1
MAP00350_Tyrosine_metabolism	0.683	1
MAP00590_Prostaglandin_and_leukotriene_metabolism	0.731	1
TCA_HG-U133A_probes	0.757	1
INS_HG-U133A_probes	0.805	1
MAP00670_One_carbon_pool_by_folate	0.868	1
c16_U133_probes	0.882	1
c11_U133_probes	0.888	1
c4_U133_probes	0.908	1
c24_U133_probes	0.908	1
c30_U133_probes	0.938	1

Continue ... ...

Top Pathways	NP	AP
c2_U133_probes	0.956	1
c14_U133_probes	0.958	1
c8_U133_probes	0.96	1
c31_U133_probes	0.97	1
MAP00480_Glutathione_metabolism	0.982	1
SAFE		
MAP00561_Glycerolipid_metabolism	0.011	1
OXPHOS_HG-U133A_probes	0.021	1
MAP00500_Starch_and_sucrose_metabolism	0.034	1
MAP00240_Pyrimidine_metabolism	0.046	1
GLUCO_HG-U133A_probes	0.049	1
MAP00190_Oxidative_phosphorylation	0.0645	1
c20_U133_probes	0.0675	1
c18_U133_probes	0.073	1
human_mitoDB_6_2002_HG-U133A_probes	0.078	1
c23_U133_probes	0.093	1
MAP00251_Glutamate_metabolism	0.105	1
MAP00910_Nitrogen_metabolism	0.109	1
mitochondr_HG-U133A_probes	0.1105	1
MAP00860_Porphyrin_and_chlorophyll_metabolism	0.131	1
MAP00252_Alanine_and_aspartate_metabolism	0.133	1
MAP00710_Carbon_fixation	0.1375	1
c7_U133_probes	0.159	1
c21_U133_probes	0.16	1
c3_U133_probes	0.1725	1
MAP00340_Histidine_metabolism	0.173	1
MAP00310_Lysine_degradation	0.175	1
MAP03020_RNA_polymerase	0.176	1
MAP00120_Bile_acid_biosynthesis	0.1775	1
MAP00510_N_Glycans_biosynthesis	0.186	1
c10_U133_probes	0.189	1
c27_U133_probes	0.189	1
MAP00052_Galactose_metabolism	0.1955	1
MAP00030_Pentose_phosphate_pathway	0.1965	1
MAP00220_Urea_cycle_and_metabolism_of_amino_groups	0.2145	1
MAP00330_Arginine_and_proline_metabolism	0.2225	1
MAP00620_Pyruvate_metabolism	0.224	1
c25_U133_probes	0.227	1
c33_U133_probes	0.2425	1
GLYCOL_HG-U133A_probes	0.2685	1
MAP00230_Purine_metabolism	0.2845	1
FA_HG-U133A_probes	0.292	1
MAP00010_Glycolysis_Gluconeogenesis	0.3175	1
	Continue	

Top Pathways	NP	AP
MAP00260_Glycine_serine_and_threonine_metabolism	0.3325	1
MAP00051_Fructose_and_mannose_metabolism	0.3335	1
GLYCOGEN_HG-133A_probes	0.336	1
c26_U133_probes	0.345	1
MAP00020_Citrate_cycle_TCA_cycle	0.3535	1
MAP00970_Aminoacyl_tRNA_biosynthesis	0.3565	1
c1_U133_probes	0.3605	1
MAP00650_Butanoate_metabolism	0.3715	1
MAP00193_ATP_synthesis	0.377	1
MAP00195_Photosynthesis	0.377	1
MAP03070_Type_III_secretion_system	0.377	1
c5_U133_probes	0.3835	1
c22_U133_probes	0.421	1
c12_U133_probes	0.424	1
MAP00280_Valine_leucine_and_isoleucine_degradation	0.4375	1
c35_U133_probes	0.461	1
c15_U133_probes	0.468	1
MAP00410_beta_Alanine_metabolism	0.472	1
MAP00640_Propanoate_metabolism	0.484	1
c29_U133_probes	0.4945	1
c19_U133_probes	0.516	1
c0_U133_probes	0.53	1
GO_0005739_HG-U133A_probes	0.545	1
MAP00380_Tryptophan_metabolism	0.5685	1
MAP00380_Tryptophan_metabolism	0.5685	1
c34_U133_probes	0.5935	1
c13_U133_probes	0.594	1
c9_U133_probes	0.599	1
c32_U133_probes	0.6065	1
c6_U133_probes	0.63	1
MAP00071_Fatty_acid_metabolism	0.654	1
c17_U133_probes	0.681	1
MAP00590_Prostaglandin_and_leukotriene_metabolism	0.6965	1
MAP00350_Tyrosine_metabolism	0.706	1
MAP00361_gamma_Hexachlorocyclohexane_degradation	0.735	1
c28_U133_probes	0.741	1
MAP00562_Inositol_phosphate_metabolism	0.743	1
INS_HG-U133A_probes	0.762	1
TCA_HG-U133A_probes	0.7745	1
c11_U133_probes	0.7755	1
c16_U133_probes	0.813	1
MAP00670_One_carbon_pool_by_folate	0.822	1
c24_U133_probes	0.8565	1
c4_U133_probes	0.9375	1
		Continue ... ...

Top Pathways	NP	AP
c8_U133_probes	0.9385	1
c30_U133_probes	0.941	1
c2_U133_probes	0.95	1
c31_U133_probes	0.9525	1
c14_U133_probes	0.964	1
MAP00480_Glutathione_metabolism	0.9835	1
GlobalTest		
MAP00252_Alanine_and_aspartate_metabolism	0.106	1
OXPHOS_HG-U133A_probes	0.123	1
c23_U133_probes	0.14	1
c25_U133_probes	0.144	1
GLUCO_HG-U133A_probes	0.144	1
c18_U133_probes	0.19	1
MAP00220_Urea_cycle_and_metabolism_of_amino_groups	0.192	1
MAP00620_Pyruvate_metabolism	0.198	1
FA_HG-U133A_probes	0.236	1
MAP00120_Bile_acid_biosynthesis	0.238	1
MAP00020_Citrate_cycle_TCA_cycle	0.248	1
MAP00500_Starch_and_sucrose_metabolism	0.254	1
MAP00561_Glycerolipid_metabolism	0.26	1
c19_U133_probes	0.261	1
MAP00190_Oxidative_phosphorylation	0.264	1
MAP00710_Carbon_fixation	0.269	1
c26_U133_probes	0.28	1
MAP00910_Nitrogen_metabolism	0.296	1
c20_U133_probes	0.302	1
c1_U133_probes	0.304	1
MAP00071_Fatty_acid_metabolism	0.331	1
c35_U133_probes	0.335	1
MAP00860_Porphyrin_and_chlorophyll_metabolism	0.335	1
c4_U133_probes	0.338	1
MAP03020_RNA_polymerase	0.346	1
MAP00310_Lysine_degradation	0.36	1
MAP00340_Histidine_metabolism	0.366	1
MAP00330_Arginine_and_proline_metabolism	0.38	1
c32_U133_probes	0.381	1
MAP00052_Galactose_metabolism	0.383	1
c28_U133_probes	0.384	1
c22_U133_probes	0.391	1
c7_U133_probes	0.4	1
MAP00970_Aminoacyl_tRNA_biosynthesis	0.4	1
MAP00410_beta_Alanine_metabolism	0.41	1
c3_U133_probes	0.414	1
	Continue	

Top Pathways	NP	AP
human_mitoDB_6_2002_HG-U133A_probes	0.428	1
c0_U133_probes	0.429	1
MAP00380_Tryptophan_metabolism	0.43	1
MAP00380_Tryptophan_metabolism	0.43	1
c27_U133_probes	0.436	1
GO_0005739_HG-U133A_probes	0.436	1
mitochondr_HG-U133A_probes	0.446	1
MAP00640_Propanoate_metabolism	0.45	1
MAP00240_Pyrimidine_metabolism	0.458	1
c33_U133_probes	0.464	1
MAP00562_Inositol_phosphate_metabolism	0.467	1
c21_U133_probes	0.468	1
MAP00260_Glycine_serine_and_threonine_metabolism	0.473	1
MAP00251_Glutamate_metabolism	0.476	1
MAP00361_gamma_Hexachlorocyclohexane_degradation	0.478	1
MAP00650_Butanoate_metabolism	0.484	1
MAP00510_N_Glycans_biosynthesis	0.508	1
c34_U133_probes	0.51	1
MAP00030_Pentose_phosphate_pathway	0.525	1
MAP00010_Glycolysis_Gluconeogenesis	0.528	1
c12_U133_probes	0.537	1
MAP00350_Tyrosine_metabolism	0.538	1
c6_U133_probes	0.542	1
c15_U133_probes	0.546	1
MAP00230_Purine_metabolism	0.568	1
c13_U133_probes	0.574	1
c29_U133_probes	0.578	1
GLYCOL_HG-U133A_probes	0.59	1
c10_U133_probes	0.61	1
c2_U133_probes	0.616	1
MAP00670_One_carbon_pool_by_folate	0.626	1
MAP00051_Fructose_and_mannose_metabolism	0.628	1
MAP00193_ATP_synthesis	0.629	1
MAP00195_Photosynthesis	0.629	1
MAP03070_Type_III_secretion_system	0.629	1
MAP00280_Valine_leucine_and_isoleucine_degradation	0.658	1
c17_U133_probes	0.676	1
c16_U133_probes	0.687	1
c9_U133_probes	0.692	1
c14_U133_probes	0.724	1
c8_U133_probes	0.736	1
c5_U133_probes	0.746	1
MAP00590_Prostaglandin_and_leukotriene_metabolism	0.764	1
c30_U133_probes	0.766	1
		Continue ... ...

Top Pathways	NP	AP
c31_U133_probes	0.786	1
TCA_HG-U133A_probes	0.796	1
c24_U133_probes	0.815	1
INS_HG-U133A_probes	0.83	1
GLYCOGEN_HG-133A_probes	0.836	1
c11_U133_probes	0.902	1
MAP00480_Glutathione_metabolism	0.959	1
PCOT2		
c20_U133_probes	0.0315	1
OXPHOS_HG-U133A_probes	0.045	1
MAP00190_Oxidative_phosphorylation	0.061	1
FA_HG-U133A_probes	0.0855	1
c28_U133_probes	0.093	1
MAP00500_Starch_and_sucrose_metabolism	0.0945	1
MAP00252_Alanine_and_aspartate_metabolism	0.111	1
c18_U133_probes	0.131	1
MAP00052_Galactose_metabolism	0.143	1
c3_U133_probes	0.145	1
MAP00220_Urea_cycle_and_metabolism_of_amino_groups	0.1515	1
MAP00020_Citrate_cycle_TCA_cycle	0.1585	1
c19_U133_probes	0.1605	1
MAP00561_Glycerolipid_metabolism	0.165	1
MAP00260_Glycine_serine_and_threonine_metabolism	0.223	1
MAP00510_N_Glycans_biosynthesis	0.252	1
c21_U133_probes	0.2525	1
c23_U133_probes	0.254	1
c6_U133_probes	0.2685	1
GLUCO_HG-U133A_probes	0.2695	1
c8_U133_probes	0.274	1
MAP00640_Propanoate_metabolism	0.283	1
c1_U133_probes	0.29	1
c30_U133_probes	0.298	1
c4_U133_probes	0.311	1
c9_U133_probes	0.326	1
c15_U133_probes	0.329	1
MAP00010_Glycolysis_Gluconeogenesis	0.332	1
MAP00120_Bile_acid_biosynthesis	0.3345	1
MAP00410_beta_Alanine_metabolism	0.337	1
MAP03020_RNA_polymerase	0.355	1
c5_U133_probes	0.3655	1
MAP00361_gamma_Hexachlorocyclohexane_degradation	0.37	1
MAP00970_Aminoacyl_tRNA_biosynthesis	0.3895	1
MAP00710_Carbon_fixation	0.402	1
	Continue	

Top Pathways	NP	AP
c25_U133_probes	0.4045	1
MAP00620_Pyruvate_metabolism	0.418	1
c31_U133_probes	0.426	1
MAP00051_Fructose_and_mannose_metabolism	0.4315	1
c33_U133_probes	0.454	1
MAP00310_Lysine_degradation	0.4735	1
MAP00860_Porphyrin_and_chlorophyll_metabolism	0.478	1
MAP00280_Valine_leucine_and_isoleucine_degradation	0.483	1
MAP00562_Inositol_phosphate_metabolism	0.4875	1
MAP00340_Histidine_metabolism	0.496	1
MAP00193_ATP_synthesis	0.5085	1
MAP00195_Photosynthesis	0.5085	1
MAP03070_Type_III_secretion_system	0.5085	1
TCA_HG-U133A_probes	0.516	1
MAP00350_Tyrosine_metabolism	0.5285	1
GO_0005739_HG-U133A_probes	0.541	1
c10_U133_probes	0.549	1
c7_U133_probes	0.553	1
MAP00380_Tryptophan_metabolism	0.5585	1
MAP00380_Tryptophan_metabolism	0.5585	1
MAP00330_Arginine_and_proline_metabolism	0.585	1
MAP00910_Nitrogen_metabolism	0.5985	1
MAP00071_Fatty_acid_metabolism	0.605	1
MAP00251_Glutamate_metabolism	0.6145	1
c2_U133_probes	0.649	1
mitochondr_HG-U133A_probes	0.6535	1
MAP00240_Pyrimidine_metabolism	0.665	1
MAP00030_Pentose_phosphate_pathway	0.6805	1
c26_U133_probes	0.6895	1
MAP00670_One_carbon_pool_by_folate	0.6945	1
human_mitoDB_6_2002_HG-U133A_probes	0.7445	1
c35_U133_probes	0.7505	1
GLYCOL_HG-U133A_probes	0.7515	1
MAP00590_Prostaglandin_and_leukotriene_metabolism	0.7645	1
MAP00650_Butanoate_metabolism	0.771	1
c29_U133_probes	0.8055	1
c32_U133_probes	0.807	1
c11_U133_probes	0.819	1
c24_U133_probes	0.8225	1
c16_U133_probes	0.843	1
c0_U133_probes	0.8545	1
c12_U133_probes	0.8665	1
c27_U133_probes	0.9015	1
c22_U133_probes	0.921	1
	Continue	

Top Pathways	NP	AP
MAP00230_Purine_metabolism	0.9245	1
GLYCOGEN_HG-133A_probes	0.9295	1
MAP00480_Glutathione_metabolism	0.949	1
c13_U133_probes	0.9605	1
INS_HG-U133A_probes	0.968	1
c34_U133_probes	0.9755	1
c17_U133_probes	0.9805	1
c14_U133_probes	0.991	1
sigPathway		
c22_U133_probes	0.012	1
c29_U133_probes	0.014	1
OXPHOS_HG-U133A_probes	0.014	1
c25_U133_probes	0.032	1
MAP00252_Alanine_and_aspartate_metabolism	0.088	1
MAP00030_Pentose_phosphate_pathway	0.108	1
c18_U133_probes	0.116	1
MAP00190_Oxidative_phosphorylation	0.118	1
MAP00500_Starch_and_sucrose_metabolism	0.145	1
c10_U133_probes	0.148	1
c23_U133_probes	0.177	1
MAP00910_Nitrogen_metabolism	0.194	1
MAP00120_Bile_acid_biosynthesis	0.195	1
MAP00251_Glutamate_metabolism	0.205	1
MAP00480_Glutathione_metabolism	0.213	1
c21_U133_probes	0.221	1
MAP00330_Arginine_and_proline_metabolism	0.234	1
c20_U133_probes	0.25	1
MAP00561_Glycerolipid_metabolism	0.251	1
MAP00350_Tyrosine_metabolism	0.258	1
MAP00010_Glycolysis_Gluconeogenesis	0.274	1
mitochondr_HG-U133A_probes	0.278	1
c33_U133_probes	0.28	1
c1_U133_probes	0.291	1
human_mitoDB_6_2002_HG-U133A_probes	0.295	1
MAP00860_Porphyrin_and_chlorophyll_metabolism	0.296	1
c3_U133_probes	0.308	1
c19_U133_probes	0.317	1
GLYCOL_HG-U133A_probes	0.32	1
c32_U133_probes	0.321	1
GLYCOGEN_HG-133A_probes	0.321	1
MAP00590_Prostaglandin_and_leukotriene_metabolism	0.355	1
GLUCO_HG-U133A_probes	0.379	1
MAP00240_Pyrimidine_metabolism	0.394	1

Continue ... ...

Top Pathways	NP	AP
MAP00510_N_Glycans_biosynthesis	0.397	1
MAP00052_Galactose_metabolism	0.403	1
MAP00051_Fructose_and_mannose_metabolism	0.428	1
c6_U133_probes	0.433	1
MAP00260_Glycine_serine_and_threonine_metabolism	0.465	1
c13_U133_probes	0.483	1
c14_U133_probes	0.487	1
c27_U133_probes	0.546	1
MAP00970_Aminoacyl_tRNA_biosynthesis	0.556	1
c30_U133_probes	0.594	1
c2_U133_probes	0.597	1
MAP00230_Purine_metabolism	0.611	1
FA_HG-U133A_probes	0.612	1
MAP00562_Inositol_phosphate_metabolism	0.616	1
c34_U133_probes	0.634	1
MAP00071_Fatty_acid_metabolism	0.636	1
c35_U133_probes	0.645	1
MAP00220_Urea_cycle_and_metabolism_of_amino_groups	0.657	1
MAP00310_Lysine_degradation	0.677	1
MAP03020_RNA_polymerase	0.684	1
c28_U133_probes	0.685	1
INS_HG-U133A_probes	0.695	1
MAP00340_Histidine_metabolism	0.696	1
MAP00710_Carbon_fixation	0.697	1
c16_U133_probes	0.708	1
c5_U133_probes	0.72	1
MAP00020_Citrate_cycle_TCA_cycle	0.731	1
MAP00620_Pyruvate_metabolism	0.743	1
c26_U133_probes	0.759	1
GO_0005739_HG-U133A_probes	0.762	1
c7_U133_probes	0.774	1
c9_U133_probes	0.775	1
c11_U133_probes	0.796	1
c24_U133_probes	0.803	1
TCA_HG-U133A_probes	0.822	1
MAP00361_gamma_Hexachlorocyclohexane_degradation	0.833	1
MAP00640_Propanoate_metabolism	0.833	1
MAP00193_ATP_synthesis	0.834	1
MAP00195_Photosynthesis	0.834	1
MAP03070_Type_III_secretion_system	0.834	1
c17_U133_probes	0.863	1
c12_U133_probes	0.864	1
c15_U133_probes	0.865	1
MAP00380_Tryptophan_metabolism	0.869	1
	Continue	

Top Pathways	NP	AP
MAP00380_Tryptophan_metabolism	0.869	1
c0_U133_probes	0.891	1
MAP00410_beta_Alanine_metabolism	0.912	1
MAP00650_Butanoate_metabolism	0.934	1
MAP00280_Valine_leucine_and_isoleucine_degradation	0.938	1
c31_U133_probes	0.945	1
c4_U133_probes	0.958	1
c8_U133_probes	0.974	1
MAP00670_One_carbon_pool_by_folate	0.999	1
