# Supplementary material for: Microarray-based gene set analysis: a comparison of current methods
Source: BMC Bioinformatics. 2008 Nov 27;9:502. doi: 10.1186/1471-2105-9-502 (PMC2607289; doi:10.1186/1471-2105-9-502)
Supplement: Additional file 5 — Application of gene set analysis methods to leukemia data[22](all gene sets). Ranked (by p-value) gene sets produced by each of the six analysis methods. NP indicates the nominal p-values and AP indicates the FDR adjusted p-values. [file 1471-2105-9-502-S5.pdf]

Additional file 2 - The full table of results on Golub's data set. Ranked (by p-value) gene sets produced by each of the five analysis methods. NP indicates the nominal p-values and AP indicates the FDR adjusted p-values.

Top Pathways	NP	AP
GSEA-Category		
Glycolysis / Gluconeogenesis	<5e-05	<5e-05
Focal adhesion	<5e-05	<5e-05
Tight junction	<5e-05	<5e-05
Leukocyte transendothelial migration	<5e-05	<5e-05
Regulation of actin cytoskeleton	<5e-05	<5e-05
Hematopoietic cell lineage	<5e-05	<5e-05
MAPK signaling pathway	<5e-05	<5e-05
Natural killer cell mediated cytotoxicity	<5e-05	<5e-05
Cytokine-cytokine receptor interaction	<5e-05	<5e-05
Tyrosine metabolism	<5e-05	<5e-05
Cell adhesion molecules (CAMs)	<5e-05	<5e-05
Apoptosis	<5e-05	<5e-05
Wnt signaling pathway	<5e-05	<5e-05
Histidine metabolism	<5e-05	<5e-05
Glutathione metabolism	<5e-05	<5e-05
Arachidonic acid metabolism	<5e-05	<5e-05
Fc epsilon RI signaling pathway	<5e-05	<5e-05
GnRH signaling pathway	<5e-05	<5e-05
Adipocytokine signaling pathway	<5e-05	<5e-05
Epithelial cell signaling in Helicobacter pylori infection	<5e-05	<5e-05
VEGF signaling pathway	<5e-05	<5e-05
B cell receptor signaling pathway	<5e-05	<5e-05
Galactose metabolism	<5e-05	<5e-05
Glycerolipid metabolism	<5e-05	<5e-05
Aminosugars metabolism	<5e-05	<5e-05
Metabolism of xenobiotics by cytochrome P450	<5e-05	<5e-05
Jak-STAT signaling pathway	<5e-05	0.002
Colorectal cancer	<5e-05	0.002
Glycerophospholipid metabolism	<5e-05	0.002
Glycan structures - degradation	<5e-05	0.002
Toll-like receptor signaling pathway	<5e-05	0.003
Pathogenic Escherichia coli infection - EHEC	<5e-05	0.003
Pathogenic Escherichia coli infection - EPEC	<5e-05	0.003
Cell cycle	<5e-05	0.003
Phenylalanine metabolism	<5e-05	0.003
Complement and coagulation cascades	<5e-05	0.003
Pentose phosphate pathway	<5e-05	0.003
Phosphatidylinositol signaling system	<5e-05	0.003
Long-term depression	<5e-05	0.003
	Continue	

Top Pathways	NP	AP
Carbon fixation	<5e-05	0.003
Insulin signaling pathway	<5e-05	0.004
Calcium signaling pathway	<5e-05	0.004
Purine metabolism	<5e-05	0.004
Starch and sucrose metabolism	<5e-05	0.004
Gap junction	<5e-05	0.004
N-Glycan degradation	<5e-05	0.004
Glycosaminoglycan degradation	<5e-05	0.004
Maturity onset diabetes of the young	<5e-05	0.006
T cell receptor signaling pathway	<5e-05	0.007
Fructose and mannose metabolism	<5e-05	0.007
Amyotrophic lateral sclerosis (ALS)	<5e-05	0.007
Pyrimidine metabolism	0.001	0.009
Sphingolipid metabolism	0.001	0.009
Cell Communication	0.001	0.01
Pyruvate metabolism	0.001	0.01
Type II diabetes mellitus	0.001	0.011
Antigen processing and presentation	0.001	0.011
TGF-beta signaling pathway	0.001	0.012
Adherens junction	0.001	0.014
Inositol phosphate metabolism	0.001	0.014
Bile acid biosynthesis	0.001	0.015
Axon guidance	0.002	0.021
Cholera - Infection	0.002	0.025
Limonene and pinene degradation	0.003	0.028
Tryptophan metabolism	0.003	0.029
Androgen and estrogen metabolism	0.003	0.03
Basal transcription factors	0.003	0.035
Neuroactive ligand-receptor interaction	0.004	0.041
ECM-receptor interaction	0.004	0.041
Methane metabolism	0.004	0.042
1- and 2-Methylnaphthalene degradation	0.005	0.048
Taste transduction	0.005	0.049
N-Glycan biosynthesis	0.006	0.054
Selenoamino acid metabolism	0.007	0.062
Fatty acid metabolism	0.007	0.066
Long-term potentiation	0.008	0.074
Glycine serine and threonine metabolism	0.009	0.078
PPAR signaling pathway	0.009	0.081
beta-Alanine metabolism	0.013	0.111
Prion disease	0.013	0.111
Arginine and proline metabolism	0.014	0.116
Pentose and glucuronate interconversions	0.014	0.116
Alkaloid biosynthesis II	0.015	0.12
	Continue	

Top Pathways	NP	AP
Nitrogen metabolism	0.015	0.12
Urea cycle and metabolism of amino groups	0.015	0.12
Type I diabetes mellitus	0.017	0.131
Biosynthesis of steroids	0.02	0.155
Glycan structures - biosynthesis 1	0.021	0.164
Proteasome	0.022	0.165
Valine leucine and isoleucine degradation	0.024	0.186
Neurodegenerative Disorders	0.027	0.201
Linoleic acid metabolism	0.027	0.201
Hedgehog signaling pathway	0.03	0.218
Huntington's disease	0.034	0.247
Propanoate metabolism	0.035	0.252
DNA polymerase	0.038	0.272
Regulation of autophagy	0.039	0.273
Glycan structures - biosynthesis 2	0.04	0.276
RNA polymerase	0.04	0.276
Dentatorubropallidoluysian atrophy (DRPLA)	0.043	0.293
Glycosphingolipid biosynthesis - neo-lactoseries	0.048	0.323
Alzheimer's disease	0.049	0.323
Notch signaling pathway	0.049	0.323
Folate biosynthesis	0.055	0.359
Dorso-ventral axis formation	0.06	0.387
Lysine degradation	0.068	0.439
Citrate cycle (TCA cycle)	0.071	0.453
Benzoate degradation via CoA ligation	0.074	0.465
Oxidative phosphorylation	0.082	0.515
C21-Steroid hormone metabolism	0.104	0.647
Olfactory transduction	0.107	0.661
mTOR signaling pathway	0.148	0.905
Butanoate metabolism	0.15	0.906
Aminoacyl-tRNA biosynthesis	0.177	1
gamma-Hexachlorocyclohexane degradation	0.188	1
Glutamate metabolism	0.198	1
Alanine and aspartate metabolism	0.209	1
Riboflavin metabolism	0.232	1
Nicotinate and nicotinamide metabolism	0.233	1
SNARE interactions in vesicular transport	0.417	1
Cysteine metabolism	0.566	1
ABC transporters - General	0.6	1
One carbon pool by folate	0.664	1
Ribosome	0.765	1
Porphyrin and chlorophyll metabolism	0.81	1
Ubiquitin mediated proteolysis	0.951	1

Continue ... ...

Top Pathways	NP	AP
GSEA-limma		
Hematopoietic cell lineage	<5e-05	0.034
B cell receptor signaling pathway	<5e-05	0.034
Glutathione metabolism	0.017	1
Glycolysis / Gluconeogenesis	0.025	1
Natural killer cell mediated cytotoxicity	0.028	1
Leukocyte transendothelial migration	0.036	1
Cell cycle	0.039	1
Proteasome	0.044	1
Galactose metabolism	0.053	1
Pentose phosphate pathway	0.056	1
Arachidonic acid metabolism	0.069	1
Aminosugars metabolism	0.077	1
Pathogenic Escherichia coli infection - EPEC	0.077	1
Pathogenic Escherichia coli infection - EHEC	0.078	1
Histidine metabolism	0.081	1
Pyrimidine metabolism	0.089	1
N-Glycan degradation	0.094	1
Cell adhesion molecules (CAMs)	0.099	1
VEGF signaling pathway	0.106	1
Glycan structures - degradation	0.122	1
Colorectal cancer	0.122	1
Wnt signaling pathway	0.127	1
Phenylalanine metabolism	0.137	1
Amyotrophic lateral sclerosis (ALS)	0.138	1
Carbon fixation	0.141	1
Glycerolipid metabolism	0.141	1
Glycosaminoglycan degradation	0.143	1
Pyruvate metabolism	0.155	1
Epithelial cell signaling in Helicobacter pylori infection	0.163	1
Metabolism of xenobiotics by cytochrome P450	0.165	1
Tight junction	0.178	1
Glycerophospholipid metabolism	0.184	1
Fructose and mannose metabolism	0.194	1
Limonene and pinene degradation	0.215	1
Tyrosine metabolism	0.222	1
Methane metabolism	0.23	1
Cholera - Infection	0.242	1
Fc epsilon RI signaling pathway	0.247	1
Sphingolipid metabolism	0.251	1
Purine metabolism	0.252	1
Adipocytokine signaling pathway	0.256	1
N-Glycan biosynthesis	0.264	1
Maturity onset diabetes of the young	0.274	1

Continue ... ...

Top Pathways	NP	AP
Prion disease	0.282	1
Starch and sucrose metabolism	0.287	1
Selenoamino acid metabolism	0.303	1
Basal transcription factors	0.304	1
Phosphatidylinositol signaling system	0.341	1
Antigen processing and presentation	0.35	1
Focal adhesion	0.352	1
1- and 2-Methylnaphthalene degradation	0.352	1
Taste transduction	0.361	1
GnRH signaling pathway	0.364	1
Pentose and glucuronate interconversions	0.377	1
Alkaloid biosynthesis II	0.381	1
Biosynthesis of steroids	0.391	1
Tryptophan metabolism	0.402	1
RNA polymerase	0.406	1
Type II diabetes mellitus	0.419	1
Regulation of actin cytoskeleton	0.43	1
Apoptosis	0.436	1
Inositol phosphate metabolism	0.442	1
Jak-STAT signaling pathway	0.446	1
Linoleic acid metabolism	0.461	1
Androgen and estrogen metabolism	0.47	1
Fatty acid metabolism	0.475	1
beta-Alanine metabolism	0.478	1
DNA polymerase	0.481	1
Bile acid biosynthesis	0.511	1
Benzoate degradation via CoA ligation	0.518	1
Dentatorubropallidoluysian atrophy (DRPLA)	0.521	1
Glycosphingolipid biosynthesis - neo-lactoseries	0.531	1
Adherens junction	0.532	1
Type I diabetes mellitus	0.535	1
Propanoate metabolism	0.536	1
Complement and coagulation cascades	0.539	1
Nitrogen metabolism	0.548	1
Glycine serine and threonine metabolism	0.551	1
TGF-beta signaling pathway	0.559	1
Oxidative phosphorylation	0.572	1
T cell receptor signaling pathway	0.598	1
Cytokine-cytokine receptor interaction	0.61	1
Urea cycle and metabolism of amino groups	0.611	1
Gap junction	0.622	1
Folate biosynthesis	0.636	1
Long-term depression	0.636	1
C21-Steroid hormone metabolism	0.644	1

Continue ... ...

Top Pathways	NP	AP
Citrate cycle (TCA cycle)	0.652	1
Insulin signaling pathway	0.667	1
Toll-like receptor signaling pathway	0.677	1
Regulation of autophagy	0.688	1
Glycan structures - biosynthesis 1	0.696	1
Calcium signaling pathway	0.697	1
Alzheimer's disease	0.732	1
Dorso-ventral axis formation	0.738	1
Valine leucine and isoleucine degradation	0.745	1
Neurodegenerative Disorders	0.747	1
Lysine degradation	0.757	1
Axon guidance	0.758	1
Glycan structures - biosynthesis 2	0.759	1
Riboflavin metabolism	0.76	1
Long-term potentiation	0.763	1
Hedgehog signaling pathway	0.775	1
Huntington's disease	0.778	1
Notch signaling pathway	0.788	1
gamma-Hexachlorocyclohexane degradation	0.8	1
Olfactory transduction	0.802	1
Aminoacyl-tRNA biosynthesis	0.805	1
Cell Communication	0.815	1
Arginine and proline metabolism	0.82	1
PPAR signaling pathway	0.857	1
Nicotinate and nicotinamide metabolism	0.859	1
ECM-receptor interaction	0.863	1
MAPK signaling pathway	0.872	1
SNARE interactions in vesicular transport	0.873	1
Cysteine metabolism	0.886	1
Butanoate metabolism	0.896	1
One carbon pool by folate	0.919	1
Alanine and aspartate metabolism	0.935	1
mTOR signaling pathway	0.936	1
Porphyrin and chlorophyll metabolism	0.984	1
Neuroactive ligand-receptor interaction	0.992	1
Ubiquitin mediated proteolysis	0.992	1
ABC transporters - General	0.994	1
Glutamate metabolism	1	1
Ribosome	1	1
SAFE		
Natural killer cell mediated cytotoxicity	0.0052	1
Glycolysis / Gluconeogenesis	0.00835	1
Galactose metabolism	0.0128	1
	Continue	

Top Pathways	NP	AP
Pyrimidine metabolism	0.0333	1
Cell cycle	0.0353	1
Arachidonic acid metabolism	0.0354	1
Leukocyte transendothelial migration	0.0406	1
Amyotrophic lateral sclerosis (ALS)	0.0429	1
Hematopoietic cell lineage	0.0458	1
B cell receptor signaling pathway	0.0461	1
Pentose phosphate pathway	0.05075	1
N-Glycan biosynthesis	0.06215	1
Purine metabolism	0.08095	1
Colorectal cancer	0.0821	1
Histidine metabolism	0.09845	1
Pyruvate metabolism	0.1064	1
Carbon fixation	0.1064	1
Aminosugars metabolism	0.11935	1
Wnt signaling pathway	0.12915	1
Pathogenic Escherichia coli infection - EHEC	0.1301	1
Pathogenic Escherichia coli infection - EPEC	0.1301	1
Maturity onset diabetes of the young	0.1427	1
Cholera - Infection	0.14395	1
Tight junction	0.144	1
Proteasome	0.1453	1
Cell adhesion molecules (CAMs)	0.1482	1
Adipocytokine signaling pathway	0.1534	1
Fructose and mannose metabolism	0.16245	1
1- and 2-Methylnaphthalene degradation	0.19185	1
Glycerolipid metabolism	0.2006	1
Glycerophospholipid metabolism	0.20625	1
RNA polymerase	0.21085	1
Focal adhesion	0.2128	1
Adherens junction	0.2171	1
Glycosaminoglycan degradation	0.22315	1
N-Glycan degradation	0.22515	1
VEGF signaling pathway	0.24065	1
Limonene and pinene degradation	0.2446	1
Glycan structures - degradation	0.25825	1
Basal transcription factors	0.26125	1
Tyrosine metabolism	0.26335	1
Starch and sucrose metabolism	0.26505	1
Prion disease	0.26945	1
Phenylalanine metabolism	0.27335	1
Epithelial cell signaling in Helicobacter pylori infection	0.2914	1
Sphingolipid metabolism	0.2944	1
Phosphatidylinositol signaling system	0.3111	1

Continue ... ...

Top Pathways	NP	AP
Selenoamino acid metabolism	0.31545	1
Dentatorubropallidoluysian atrophy (DRPLA)	0.31735	1
Antigen processing and presentation	0.32145	1
DNA polymerase	0.3288	1
Androgen and estrogen metabolism	0.33635	1
TGF-beta signaling pathway	0.3371	1
Glutathione metabolism	0.33835	1
Regulation of actin cytoskeleton	0.34925	1
Taste transduction	0.36135	1
Type II diabetes mellitus	0.3652	1
Alkaloid biosynthesis II	0.37895	1
Fatty acid metabolism	0.3826	1
GnRH signaling pathway	0.3927	1
Inositol phosphate metabolism	0.4031	1
Glycan structures - biosynthesis 1	0.41345	1
Glycosphingolipid biosynthesis - neo-lactoseries	0.4138	1
Apoptosis	0.42105	1
Type I diabetes mellitus	0.4238	1
Fc epsilon RI signaling pathway	0.4241	1
Nitrogen metabolism	0.43305	1
Methane metabolism	0.44955	1
T cell receptor signaling pathway	0.4516	1
Pentose and glucuronate interconversions	0.4526	1
Tryptophan metabolism	0.45785	1
Glycine serine and threonine metabolism	0.4666	1
Gap junction	0.4689	1
Biosynthesis of steroids	0.4935	1
Bile acid biosynthesis	0.50265	1
Insulin signaling pathway	0.5267	1
Linoleic acid metabolism	0.52755	1
Cytokine-cytokine receptor interaction	0.5355	1
Notch signaling pathway	0.5355	1
Benzoate degradation via CoA ligation	0.5405	1
Jak-STAT signaling pathway	0.5407	1
beta-Alanine metabolism	0.55175	1
Cell Communication	0.5569	1
Toll-like receptor signaling pathway	0.5603	1
Regulation of autophagy	0.5615	1
Dorso-ventral axis formation	0.56995	1
Glycan structures - biosynthesis 2	0.5702	1
Huntington's disease	0.58455	1
Oxidative phosphorylation	0.5939	1
Metabolism of xenobiotics by cytochrome P450	0.6361	1
C21-Steroid hormone metabolism	0.63775	1

Continue ... ...

Top Pathways	NP	AP
Propanoate metabolism	0.6416	1
Neurodegenerative Disorders	0.64215	1
Calcium signaling pathway	0.6656	1
Urea cycle and metabolism of amino groups	0.66775	1
Long-term depression	0.6771	1
ECM-receptor interaction	0.68335	1
Axon guidance	0.72595	1
Folate biosynthesis	0.7548	1
Alanine and aspartate metabolism	0.7556	1
Valine leucine and isoleucine degradation	0.7643	1
Hedgehog signaling pathway	0.7648	1
Citrate cycle (TCA cycle)	0.76895	1
Lysine degradation	0.7704	1
Complement and coagulation cascades	0.7787	1
Alzheimer's disease	0.78335	1
Cysteine metabolism	0.80175	1
Olfactory transduction	0.824	1
Aminoacyl-tRNA biosynthesis	0.83585	1
gamma-Hexachlorocyclohexane degradation	0.8387	1
SNARE interactions in vesicular transport	0.84215	1
Butanoate metabolism	0.8458	1
MAPK signaling pathway	0.8497	1
Ribosome	0.87625	1
PPAR signaling pathway	0.87825	1
Nicotinate and nicotinamide metabolism	0.88035	1
Arginine and proline metabolism	0.8813	1
Riboflavin metabolism	0.9007	1
Long-term potentiation	0.91695	1
Neuroactive ligand-receptor interaction	0.9487	1
One carbon pool by folate	0.94995	1
mTOR signaling pathway	0.96255	1
Ubiquitin mediated proteolysis	0.97405	1
Porphyrin and chlorophyll metabolism	0.97945	1
ABC transporters - General	0.98965	1
Glutamate metabolism	0.9996	1
GlobalTest		
Toll-like receptor signaling pathway	<5e-05	<5e-05
Jak-STAT signaling pathway	<5e-05	<5e-05
Focal adhesion	<5e-05	<5e-05
Tight junction	<5e-05	<5e-05
Leukocyte transendothelial migration	<5e-05	<5e-05
Regulation of actin cytoskeleton	<5e-05	<5e-05
Hematopoietic cell lineage	<5e-05	<5e-05
	Continue	

Top Pathways	NP	AP
MAPK signaling pathway	<5e-05	<5e-05
Natural killer cell mediated cytotoxicity	<5e-05	<5e-05
Cytokine-cytokine receptor interaction	<5e-05	<5e-05
Calcium signaling pathway	<5e-05	<5e-05
Cell adhesion molecules (CAMs)	<5e-05	<5e-05
Wnt signaling pathway	<5e-05	<5e-05
Colorectal cancer	<5e-05	<5e-05
Histidine metabolism	<5e-05	<5e-05
Glutathione metabolism	<5e-05	<5e-05
Arachidonic acid metabolism	<5e-05	<5e-05
Fc epsilon RI signaling pathway	<5e-05	<5e-05
GnRH signaling pathway	<5e-05	<5e-05
Epithelial cell signaling in Helicobacter pylori infection	<5e-05	<5e-05
Complement and coagulation cascades	<5e-05	<5e-05
VEGF signaling pathway	<5e-05	<5e-05
B cell receptor signaling pathway	<5e-05	<5e-05
Glycerolipid metabolism	<5e-05	<5e-05
Phosphatidylinositol signaling system	<5e-05	<5e-05
Aminosugars metabolism	<5e-05	<5e-05
Sphingolipid metabolism	<5e-05	<5e-05
N-Glycan degradation	<5e-05	<5e-05
Metabolism of xenobiotics by cytochrome P450	<5e-05	<5e-05
Pathogenic Escherichia coli infection - EHEC	<5e-05	0.001
Pathogenic Escherichia coli infection - EPEC	<5e-05	0.001
TGF-beta signaling pathway	<5e-05	0.001
Phenylalanine metabolism	<5e-05	0.001
Methane metabolism	<5e-05	0.001
Long-term depression	<5e-05	0.001
Glycan structures - degradation	<5e-05	0.001
Cell cycle	<5e-05	0.002
Type II diabetes mellitus	<5e-05	0.002
Adipocytokine signaling pathway	<5e-05	0.002
Cell Communication	<5e-05	0.002
Glycerophospholipid metabolism	<5e-05	0.002
Gap junction	<5e-05	0.002
Antigen processing and presentation	<5e-05	0.002
Axon guidance	<5e-05	0.003
Purine metabolism	<5e-05	0.003
Folate biosynthesis	<5e-05	0.003
Neuroactive ligand-receptor interaction	<5e-05	0.004
Starch and sucrose metabolism	<5e-05	0.005
Glycosaminoglycan degradation	<5e-05	0.005
Inositol phosphate metabolism	<5e-05	0.005
PPAR signaling pathway	0.001	0.01
		Continue ... ...

Top Pathways	NP	AP
Pyrimidine metabolism	0.001	0.01
Adherens junction	0.001	0.011
Androgen and estrogen metabolism	0.001	0.014
ECM-receptor interaction	0.001	0.014
Long-term potentiation	0.001	0.014
Basal transcription factors	0.001	0.014
Hedgehog signaling pathway	0.001	0.014
Pentose and glucuronate interconversions	0.001	0.014
Pyruvate metabolism	0.001	0.015
Carbon fixation	0.001	0.016
Taste transduction	0.001	0.016
Glycolysis / Gluconeogenesis	0.002	0.016
Proteasome	0.002	0.021
Riboflavin metabolism	0.002	0.026
Biosynthesis of steroids	0.003	0.026
Alkaloid biosynthesis II	0.003	0.027
Oxidative phosphorylation	0.003	0.027
Glycan structures - biosynthesis 2	0.003	0.029
Insulin signaling pathway	0.004	0.038
Tyrosine metabolism	0.005	0.047
Type I diabetes mellitus	0.005	0.047
Galactose metabolism	0.005	0.048
Nicotinate and nicotinamide metabolism	0.005	0.048
Cholera - Infection	0.005	0.048
Prion disease	0.005	0.049
1- and 2-Methylnaphthalene degradation	0.006	0.051
Fructose and mannose metabolism	0.006	0.053
Amyotrophic lateral sclerosis (ALS)	0.006	0.054
Alzheimer's disease	0.007	0.056
Pentose phosphate pathway	0.007	0.057
Glycosphingolipid biosynthesis - neo-lactoseries	0.007	0.061
Apoptosis	0.008	0.064
Linoleic acid metabolism	0.009	0.071
Neurodegenerative Disorders	0.01	0.076
Notch signaling pathway	0.011	0.086
Selenoamino acid metabolism	0.013	0.098
gamma-Hexachlorocyclohexane degradation	0.015	0.115
Dorso-ventral axis formation	0.015	0.118
DNA polymerase	0.017	0.128
Nitrogen metabolism	0.02	0.151
Maturity onset diabetes of the young	0.022	0.167
Glycan structures - biosynthesis 1	0.026	0.194
N-Glycan biosynthesis	0.028	0.202
Citrate cycle (TCA cycle)	0.029	0.206

Continue ... ...

Top Pathways	NP	AP
T cell receptor signaling pathway	0.032	0.225
One carbon pool by folate	0.032	0.227
Tryptophan metabolism	0.037	0.255
RNA polymerase	0.037	0.255
Regulation of autophagy	0.039	0.266
Olfactory transduction	0.054	0.362
Arginine and proline metabolism	0.055	0.366
Huntington's disease	0.062	0.408
Bile acid biosynthesis	0.064	0.418
Dentatorubropallidoluysian atrophy (DRPLA)	0.065	0.424
Fatty acid metabolism	0.066	0.424
Lysine degradation	0.088	0.564
Valine leucine and isoleucine degradation	0.094	0.596
mTOR signaling pathway	0.101	0.631
beta-Alanine metabolism	0.102	0.631
Propanoate metabolism	0.106	0.649
Ubiquitin mediated proteolysis	0.113	0.689
Limonene and pinene degradation	0.136	0.823
SNARE interactions in vesicular transport	0.147	0.878
Glycine serine and threonine metabolism	0.159	0.945
Butanoate metabolism	0.161	0.95
Urea cycle and metabolism of amino groups	0.171	1
C21-Steroid hormone metabolism	0.182	1
Glutamate metabolism	0.196	1
Aminoacyl-tRNA biosynthesis	0.223	1
ABC transporters - General	0.265	1
Alanine and aspartate metabolism	0.268	1
Benzoate degradation via CoA ligation	0.274	1
Porphyrin and chlorophyll metabolism	0.348	1
Ribosome	0.379	1
Cysteine metabolism	0.528	1
PCOT2		
Jak-STAT signaling pathway	<5e-05	0.001
Glycolysis Gluconeogenesis	<5e-05	0.001
Focal adhesion	<5e-05	0.001
Tight junction	<5e-05	0.001
Hematopoietic cell lineage	<5e-05	0.001
Neuroactive ligand-receptor interaction	<5e-05	0.001
Natural killer cell mediated cytotoxicity	<5e-05	0.001
Cytokine-cytokine receptor interaction	<5e-05	0.001
Cell cycle	<5e-05	0.001
Oxidative phosphorylation	<5e-05	0.001
Calcium signaling pathway	<5e-05	0.001
		Continue ... ...

Top Pathways	NP	AP
Cell adhesion molecules (CAMs)	<5e-05	0.001
Wnt signaling pathway	<5e-05	0.001
Glutathione metabolism	<5e-05	0.001
Arachidonic acid metabolism	<5e-05	0.001
Fc epsilon RI signaling pathway	<5e-05	0.001
Purine metabolism	<5e-05	0.001
Epithelial cell signaling in Helicobacter pylori infection	<5e-05	0.001
Phenylalanine metabolism	<5e-05	0.001
Folate biosynthesis	<5e-05	0.001
Complement and coagulation cascades	<5e-05	0.001
VEGF signaling pathway	<5e-05	0.001
B cell receptor signaling pathway	<5e-05	0.001
Phosphatidylinositol signaling system	<5e-05	0.001
Aminosugars metabolism	<5e-05	0.001
N-Glycan degradation	<5e-05	0.001
Glycan structures - degradation	<5e-05	0.001
Metabolism of xenobiotics by cytochrome P450	<5e-05	0.001
Leukocyte transendothelial migration	0.0001	0.002
Regulation of actin cytoskeleton	0.0001	0.002
Pathogenic Escherichia coli infection - EHEC	0.0001	0.002
Pathogenic Escherichia coli infection - EPEC	0.0001	0.002
Colorectal cancer	0.0001	0.002
GnRH signaling pathway	0.0001	0.002
Adipocytokine signaling pathway	0.0001	0.002
Methane metabolism	0.0001	0.002
Pentose phosphate pathway	0.0001	0.002
Gap junction	0.0001	0.002
Glycerolipid metabolism	0.0001	0.002
Type II diabetes mellitus	0.00015	0.002
Sphingolipid metabolism	0.00015	0.002
Galactose metabolism	0.0002	0.003
Long-term depression	0.0002	0.003
Pyrimidine metabolism	0.00025	0.004
MAPK signaling pathway	0.0003	0.004
Axon guidance	0.00035	0.005
Tyrosine metabolism	0.00035	0.005
TGF-beta signaling pathway	0.00045	0.006
Glycosaminoglycan degradation	0.0005	0.007
Adherens junction	0.00075	0.01
Glycosphingolipid biosynthesis - neo-lactoseries	0.00075	0.01
Antigen processing and presentation	0.00095	0.012
Toll-like receptor signaling pathway	0.001	0.013
Histidine metabolism	0.001	0.013
Starch and sucrose metabolism	0.00125	0.015
	Continue	

Top Pathways	NP	AP
Carbon fixation	0.0013	0.016
Inositol phosphate metabolism	0.00145	0.017
Taste transduction	0.0016	0.019
Fructose and mannose metabolism	0.00185	0.021
Glycerophospholipid metabolism	0.0023	0.026
Androgen and estrogen metabolism	0.00235	0.026
Pyruvate metabolism	0.0024	0.026
Alzheimer's disease	0.00335	0.036
Prion disease	0.0039	0.041
Riboflavin metabolism	0.00415	0.043
Pentose and glucuronate interconversions	0.00445	0.046
Basal transcription factors	0.00505	0.051
Proteasome	0.00515	0.051
Biosynthesis of steroids	0.00555	0.055
Amyotrophic lateral sclerosis (ALS)	0.00585	0.057
Hedgehog signaling pathway	0.00655	0.063
Cholera - Infection	0.0077	0.073
Alkaloid biosynthesis II	0.00885	0.082
Insulin signaling pathway	0.0096	0.088
Apoptosis	0.014	0.127
Glycan structures - biosynthesis 1	0.01605	0.144
Selenoamino acid metabolism	0.01705	0.15
Glycan structures - biosynthesis 2	0.01715	0.15
Neurodegenerative Disorders	0.01815	0.156
PPAR signaling pathway	0.0199	0.169
One carbon pool by folate	0.02045	0.17
N-Glycan biosynthesis	0.0205	0.17
Cell Communication	0.02195	0.18
1- and 2-Methylnaphthalene degradation	0.02385	0.193
Notch signaling pathway	0.02425	0.194
Dorso-ventral axis formation	0.0264	0.209
Nicotinate and nicotinamide metabolism	0.02855	0.224
Linoleic acid metabolism	0.0338	0.262
SNARE interactions in vesicular transport	0.0342	0.262
DNA polymerase	0.0362	0.274
Type I diabetes mellitus	0.0381	0.285
Lysine degradation	0.0385	0.285
Ubiquitin mediated proteolysis	0.0416	0.305
Propanoate metabolism	0.04585	0.333
ECM-receptor interaction	0.0788	0.566
gamma-Hexachlorocyclohexane degradation	0.0866	0.615
Citrate cycle (TCA cycle)	0.088	0.619
Long-term potentiation	0.09145	0.633
Glutamate metabolism	0.0919	0.633
	Continue	

Top Pathways	NP	AP
RNA polymerase	0.0943	0.643
Regulation of autophagy	0.104	0.703
beta-Alanine metabolism	0.12535	0.839
Benzoate degradation via CoA ligation	0.14445	0.957
Maturity onset diabetes of the young	0.1461	0.959
C21-Steroid hormone metabolism	0.1539	1
Urea cycle and metabolism of amino groups	0.1674	1
Nitrogen metabolism	0.18715	1
ABC transporters - General	0.1951	1
Arginine and proline metabolism	0.20785	1
mTOR signaling pathway	0.25095	1
Alanine and aspartate metabolism	0.2549	1
Limonene and pinene degradation	0.25495	1
Olfactory transduction	0.2572	1
Bile acid biosynthesis	0.29525	1
Glycine serine and threonine metabolism	0.31885	1
Valine leucine and isoleucine degradation	0.3251	1
Fatty acid metabolism	0.3506	1
Tryptophan metabolism	0.36035	1
Butanoate metabolism	0.37715	1
Aminoacyl-tRNA biosynthesis	0.44785	1
T cell receptor signaling pathway	0.4982	1
Cysteine metabolism	0.57685	1
Porphyrin and chlorophyll metabolism	0.58805	1
Huntington's disease	0.5903	1
Ribosome	0.679	1
Dentatorubropallidoluysian atrophy (DRPLA)	0.84295	1
sigPathway		
Arachidonic acid metabolism	<5e-05	0.001
Metabolism of xenobiotics by cytochrome P450	<5e-05	0.004
Glutathione metabolism	<5e-05	0.009
Cell cycle	0.0001	0.016
Starch and sucrose metabolism	0.0002	0.023
Complement and coagulation cascades	0.0002	0.023
Glycan structures - degradation	0.0003	0.03
Cytokine-cytokine receptor interaction	0.0007	0.054
B cell receptor signaling pathway	0.0007	0.054
Epithelial cell signaling in Helicobacter pylori infection	0.001	0.069
ECM-receptor interaction	0.0012	0.075
Glycosaminoglycan degradation	0.0021	0.121
Sphingolipid metabolism	0.0025	0.133
Fc epsilon RI signaling pathway	0.0035	0.165
N-Glycan degradation	0.0036	0.165
	Continue	

Top Pathways	NP	AP
DNA polymerase	0.0041	0.177
Toll-like receptor signaling pathway	0.0077	0.312
Linoleic acid metabolism	0.0086	0.326
Cell Communication	0.009	0.326
Aminosugars metabolism	0.0112	0.386
Jak-STAT signaling pathway	0.0133	0.425
Proteasome	0.0138	0.425
Focal adhesion	0.0142	0.425
Purine metabolism	0.0168	0.482
Neuroactive ligand-receptor interaction	0.0185	0.499
Urea cycle and metabolism of amino groups	0.0205	0.499
NA	0.0206	0.499
Benzoate degradation via CoA ligation	0.021	0.499
Folate biosynthesis	0.021	0.499
Colorectal cancer	0.0224	0.514
SNARE interactions in vesicular transport	0.0265	0.589
Axon guidance	0.0274	0.59
Biosynthesis of steroids	0.0293	0.612
Arginine and proline metabolism	0.0412	0.835
Glycolysis / Gluconeogenesis	0.045	0.886
Ubiquitin mediated proteolysis	0.0521	0.978
Amyotrophic lateral sclerosis (ALS)	0.0525	0.978
Porphyrin and chlorophyll metabolism	0.0555	1
Wnt signaling pathway	0.0639	1
Huntington's disease	0.0651	1
Pyrimidine metabolism	0.0674	1
GnRH signaling pathway	0.0701	1
Pyruvate metabolism	0.0702	1
Tight junction	0.0728	1
Alkaloid biosynthesis II	0.077	1
RNA polymerase	0.0816	1
Cholera - Infection	0.0827	1
Type I diabetes mellitus	0.0831	1
Methane metabolism	0.1032	1
TGF-beta signaling pathway	0.1032	1
Glycan structures - biosynthesis 1	0.1115	1
Type II diabetes mellitus	0.112	1
Aminoacyl-tRNA biosynthesis	0.1193	1
Propanoate metabolism	0.1229	1
Adherens junction	0.1276	1
Regulation of actin cytoskeleton	0.1293	1
Long-term potentiation	0.1311	1
Cysteine metabolism	0.1339	1
Selenoamino acid metabolism	0.1388	1

Continue ... ...

Top Pathways	NP	AP
Nitrogen metabolism	0.1395	1
Maturity onset diabetes of the young	0.1458	1
Antigen processing and presentation	0.1588	1
Valine, leucine and isoleucine degradation	0.1591	1
One carbon pool by folate	0.1599	1
gamma-Hexachlorocyclohexane degradation	0.1613	1
mTOR signaling pathway	0.1629	1
Pathogenic Escherichia coli infection - EHEC	0.1657	1
Pathogenic Escherichia coli infection - EPEC	0.1657	1
Citrate cycle (TCA cycle)	0.2042	1
Phenylalanine metabolism	0.2089	1
Natural killer cell mediated cytotoxicity	0.2137	1
Pentose and glucuronate interconversions	0.2206	1
Leukocyte transendothelial migration	0.2235	1
Alanine and aspartate metabolism	0.2265	1
Notch signaling pathway	0.2269	1
Calcium signaling pathway	0.2303	1
Basal transcription factors	0.2362	1
Hematopoietic cell lineage	0.2504	1
Galactose metabolism	0.2566	1
beta-Alanine metabolism	0.2754	1
Glycosphingolipid biosynthesis - neo-lactoseries	0.2769	1
VEGF signaling pathway	0.3518	1
Fructose and mannose metabolism	0.3549	1
Glycine, serine and threonine metabolism	0.3595	1
Pentose phosphate pathway	0.3737	1
Prion disease	0.380	1
Lysine degradation	0.4003	1
MAPK signaling pathway	0.4166	1
Ribosome	0.422	1
1- and 2-Methylnaphthalene degradation	0.4294	1
N-Glycan biosynthesis	0.4374	1
Tyrosine metabolism	0.4413	1
Adipocytokine signaling pathway	0.4434	1
Carbon fixation	0.4471	1
Fatty acid metabolism	0.4669	1
Bile acid biosynthesis	0.4904	1
Regulation of autophagy	0.5239	1
Butanoate metabolism	0.5542	1
Insulin signaling pathway	0.5656	1
Alzheimer's disease	0.5898	1
Inositol phosphate metabolism	0.6063	1
Oxidative phosphorylation	0.6094	1
Cell adhesion molecules (CAMs)	0.6455	1

Continue ... ...

Top Pathways	NP	AP
Nicotinate and nicotinamide metabolism	0.6592	1
Apoptosis	0.6882	1
Androgen and estrogen metabolism	0.7051	1
Long-term depression	0.7111	1
Riboflavin metabolism	0.7278	1
Glycan structures - biosynthesis 2	0.7291	1
Tryptophan metabolism	0.739	1
Taste transduction	0.7396	1
Limonene and pinene degradation	0.7425	1
Phosphatidylinositol signaling system	0.7598	1
Glutamate metabolism	0.7631	1
Hedgehog signaling pathway	0.7745	1
Neurodegenerative Disorders	0.788	1
Dentatorubropallidoluysian atrophy (DRPLA)	0.8048	1
Gap junction	0.818	1
Dorso-ventral axis formation	0.8966	1
Glycerolipid metabolism	0.9086	1
C21-Steroid hormone metabolism	0.9262	1
ABC transporters - General	0.9403	1
Glycerophospholipid metabolism	0.9499	1
PPAR signaling pathway	0.9555	1
Histidine metabolism	0.9622	1
T cell receptor signaling pathway	0.9783	1
Olfactory transduction	0.9876	1
